# Identification of a senescence-related transcriptional signature to uncover molecular subtypes and key genes in hepatocellular carcinoma

**DOI:** 10.1371/journal.pone.0311696

**Published:** 2024-10-09

**Authors:** Xiaorong He, Fahui Liu, Qiming Gong

**Affiliations:** 1 Dermatology Institute of Fuzhou, Dermatology Hospital of Fuzhou, Fuzhou, China; 2 Xiamen Cell Therapy Research Center, The First Affiliated Hospital of Xiamen University, School of Medicine, Xiamen University, Xiamen, Fujian Province, China; 3 Department of Nephrology, Affiliated Hospital of Youjiang Medical University for Nationalities, Baise, China; 4 Education Department of Guangxi Zhuang Autonomous Region, Baise Key Laboratory for Metabolic Diseases (Youjiang Medical University for Nationalities), Baise, China; Rutgers: Rutgers The State University of New Jersey, UNITED STATES OF AMERICA

## Abstract

Hepatocellular carcinoma (HCC) is a cancer caused by abnormal cell growth due to faulty signal transduction. Cells secrete tumor suppressor factors in response to potential carcinogenic signals, inducing cellular senescence (CS) as a countermeasure. However, accurately measuring CS levels in different types of tumors is challenging due to tumor heterogeneity and the lack of universal and specific CS markers. Machine learning has revealed unique molecular traits in HCC patients, leading to clinical advantages. More research is needed to understand senescence-related molecular features in these patients. In this study, the gene expression profile features of patients with HCC were analyzed by integrating single-cell RNA sequencing and bulk RNA-seq datasets from HCC samples. The analysis identified the senescence-related pathways exhibiting HCC specificity. Subsequently, genes from these pathways were used to identify senescence-related molecular subtypes in HCC, showing significant variations in biological and clinical attributes. An HCC-specific CS risk model developed in this study revealed substantial associations between the patients’ CS scores and prognosis grouping, clinical staging, immune infiltration levels, immunotherapy response, and drug sensitivity levels. Within the constructed model, *G6PD* was identified as a key gene, potentially serving as a senescence-related target in liver cancer. Molecular biology experiments demonstrated that overexpression of *G6PD* effectively promotes the proliferative, invasive, and migration capacities of HepG2 and SK-HEP-1 cells. In conclusion, this analysis offers a valuable framework for understanding senescence in HCC and introduces a new biomarker. These findings improve our understanding of senescence in HCC and have potential for future research.

## Introduction

Liver carcinoma represents a major global health challenge, of which hepatocellular carcinoma (HCC) is the predominant form. HCC is the fourth leading cause of cancer-associated mortality worldwide [1]. Sorafenib, a multi-targeted tyrosine kinase inhibitor, is pivotal in HCC management [2]. Additionally, the combined administration of atezolizumab and bevacizumab has emerged as an innovative therapeutic strategy to support the patient’s immune system and limit tumor angiogenesis and growth [3]. These therapeutic avenues underscore the importance of multi-targeted approaches in treating patients with HCC.

Despite promising results in clinical trials, the above treatments are efficacious for only a subset of patients. Thus, the situation underscores the importance of identifying the HCC molecular signatures to enable the tailoring of the most appropriate therapeutic regimens. Recent research introduced a novel cancer treatment modality that combines pro-senescence drugs with senolytics [4], which shed light on the therapeutic promise of the cellular senescence (CS) phenotype in tumor management. While identifying senescent traits across various diseases can guide clinical interventions, describing senescence features in various tumors is still being explored and investigated.

CS represents a stress response aimed at eliminating superfluous, damaged, or aberrant cells. This phenomenon involves the persistent cessation of cell proliferation accompanied by pro-inflammatory mediator secretion, which is commonly known as the senescence-associated secretory phenotype (SASP) [5]. Initial studies described the tumor-suppressive properties of CS predicated on its capacity to inhibit tumorigenic proliferation [6]. However, emerging evidence underscored the paradoxical role of senescence in promoting cancer progression through SASP-mediated intercellular interactions [7]. Specifically, the tumorigenic promotion of specific SASP components has been highlighted. For example, IL-6 and IL-8 are prevalent within the SASP [8] and are cancer cell proliferation drivers. CCL5 amplifies tumorigenic proliferation by modulating c-MYC and cyclin D1 [9]. Furthermore, CXCL5 and VEGF augmented vascular density in heterograft tumor models[10]. Beyond angiogenesis, the SASP milieu fosters cell motility and invasion, intensifying cancer cell invasive capacity and facilitating metastatic dissemination [11,12]. Concurrently, clinical trials investigating senolytics (a therapeutic cocktail comprising dasatinib and quercetin designed for selective senescent cell extermination) demonstrated potential for alleviating age-associated pathologies and extending the lifespan in experimental animals [13]. However, concerns regarding the specificity and deleterious adverse effects of senescent cell clearance via senolytics moderate their therapeutic promise [14]. Thus, the clinical benefits of senescence-based interventions currently remain elusive. Delineating senescence signatures in HCC could provide pivotal insights for therapeutic strategizing.

In this study, a preliminary analysis was conducted using both bulk RNA sequencing (RNA-seq) and single-cell RNA (scRNA) datasets to improve understanding of HCC senescence signatures. The approach successfully pinpointed the HCC-specific senescence pathways. Specific genes from these pathways were used to construct two predictive models using machine learning algorithms. These models were designed to robustly evaluate the associations between varying degrees of senescence signatures and the clinical and molecular biological traits of patients with HCC.

Collectively, this study revealed pivotal senescence markers in HCC and provided insights for constructing reliable prediction frameworks. Serendipitously, this study also discovered a novel HCC senescence marker. Overall, the findings potentially contribute to personalized therapeutic strategies and prognostic assessments. The results provide valuable insights into the underlying mechanisms of HCC development and can guide future clinical research and drug development.

## Methods and materials

### Data resources

HCC data in TPM (transcripts per million) format were obtained from the Cancer Genome Atlas (TCGA) database and accompanied by pertinent clinical phenotype information. Concurrently, an additional HCC high-throughput sequencing dataset, the HCCDB18 cohort, was downloaded from the HCCDB database. Furthermore, GSE76427 dataset expression profile data and survival information were retrieved from the Gene Expression Omnibus (GEO) database. The refined dataset comprised 365 tumor specimens and 50 adjacent non-tumor tissue samples from TCGA-LIHC (liver HCC). One hundred and fifteen tumor tissues were identified from the GSE76427 dataset, and an additional 212 tumor tissues were obtained from the HCCDB18 cohort. In addition to these datasets, HCC scRNA data (GSE149614) were acquired from the GEO database.

### Single-cell data processing

Single-cell RNA sequencing downstream analyses were conducted utilizing the Seurat R software package (v4.3.0)[15]. The single-cell dataset underwent rigorous filtering and normalization to ensure high-quality data. First, we filtered the scRNA-seq data by requiring that each gene must be expressed in a minimum of three cells, while each cell must express at least 250 genes. Subsequently, mitochondrial and rRNA content were screened with stringent criteria to ensure that gene expression levels per cell were <5000 and >100, with mitochondrial content limited to <15%. The data from 10 samples were standardized with log-normalization. High-variation genes were identified through the FindVariableFeatures function, which enabled the detection of genes exhibiting substantial variation across cells.

The FindIntegrationAnchors function was utilized for canonical correlation analysis to integrate the data from multiple samples and overcome batch effects, followed by sample integration using the IntegrateData method. To reduce dimensionality and identify key cellular features, the expression values of all genes were scaled using the ScaleData function, followed by principal component analysis (PCA) to reduce the dimension to 30. The resolution parameter for cellular clustering was set at 0.2, enabling the identification of distinct cellular subpopulations. The distribution of filtered and processed cells in a low-dimensional space was visualized using the RunTSNE function. This visualization enabled the projection of the cells onto a two-dimensional plane, yielding a visual representation of their spatial arrangement.

The resulting clusters were annotated by assigning immune cell types to each subcluster based on classical marker genes. Specifically, subclusters 2, 7, and 10 were identified as T cells (expressing CD2, CD3D, CD3E, and. CD3G), subclusters 1 and 4 represented macrophages (expressing CD163 and CD68), subcluster 11 comprised B cells (expressing CD19, CD79A, and MS4A1), subcluster 9 consisted of plasma cells (expressing CD79A and JSRP1), subcluster 12 included mast cells (expressing TPSAB1 and CPA3), subcluster 5 was identified as fibroblasts (expressing ACTA2, PDGFRB, and NOTCH3), subcluster 8 represented endothelial cells (expressing PECAM1), while subclusters 0, 3, and 6 corresponded to hepatocytes (expressing GPC3, CD24, and MDK). Specific marker genes for each subcluster were identified using the FindAllMarkers function. Differentially expressed genes were characterized using a log-fold change threshold (logFC) of 0.5 and a minimum expression percentage (Minpct) of 0.35. Copy number variation (CNV) changes in the single-cell data were predicted using the CopyKAT package [16] to discriminate tumor cells from normal cells within each sample. Furthermore, the cell senescence-related pathways were downloaded from the Gene Set Enrichment Analysis (GSEA) website. Pathway-specific scores were computed using single-sample (ss)GSEA to assess their association with malignant and non-malignant cells. Lastly, z-score normalization was applied to the enrichment scores of each pathway to improve comparability.

### Development of senescence-related molecular subtypes and risk model

Consensus clustering of gene expression profiles was conducted using the ConsensusClusterPlus package to delineate clusters characterized by distinct gene expression signatures. The partitioning around medoids (PAM) algorithm was used in conjunction with the Euclidean metric for distance calculation to enhance the robustness of the findings. Furthermore, the methodology included 500 bootstrap iterations, each incorporating 80% of the patient cohort in the training set. Cluster numbers of 2–10 were explored. Subsequently, the resultant consensus matrix and cumulative distribution function (CDF) of the consensus were scrutinized to ascertain the optimal cluster quantity. The prognostic potential of the identified gene expression patterns was assessed using Lasso and Cox regression analysis using the R glmnet package. This approach enabled the identification of a subset of relevant genes and the construction of a RiskScore model for prognostic classification. The prognostic precision of the RiskScore model was assessed by a receiver operating characteristic (ROC) analysis using the timeROC package. Assessing the area under the ROC curve (AUC) determined the discriminatory power of the model in classifying patients based on prognosis.

### Gene set source and quantification

The specific sources of the gene set used in this study are as follows: 1) 31 cell cycle progression (CCP) genes and 24 genes for tumor angiogenesis signatures were from previous studies [17,18]; 2) 27 genes related to the G1/S phase were from the Kyoto Encyclopedia of Genes and Genomes (KEGG) website; 3) hallmark gene sets (REACTOME_TELOMERE_EXTENSION_BY_TELOMERASE, HALLMARK_EPITHELIAL_MESENCHYMAL_TRANSITION, HALLMARK_G2M_CHECKPOINT, HALLMARK_INFLAMMATORY_RESPONSE, and HALLMARK_HYPOXIA), were from the GSEA website; 4) the relevant gene sets for 10 tumor-related pathways were from a previous study [19].

The gene sets mentioned earlier were calculated using single-sample (ss) GSEA to obtain corresponding scores. The immune and stromal scores were computed using the ESTIMATE algorithm [20], a computational approach that estimates the immune and stromal components present within the tumor microenvironment. These scores provide an assessment of the overall immune cell infiltration and stromal activity levels in each sample. Additionally, the immune cell scores were determined using the CIBERSORT algorithm [21], which calculates the proportions of different immune cell types within the sample. These scoring calculations were all conducted using TCGA samples.

### Cell culture and transfection

The human HCC cell line HepG2 (KCB200507YJ) was obtained from the Chinese Academy of Sciences. The HepG2 cells were cultured in RPMI 1640 medium supplemented with 10% fetal bovine serum (FBS), glutamine, and HEPES at a temperature of 37°C in a 5% CO_2_ incubator. The SK-HEP-1 cell line was procured from Servicebio (Wuhan, China). The SK-HEP-1 cells were maintained in DMEM medium supplemented with 10% FBS and 1% antibiotics, and were housed in a humidified incubator set at 37°C with 5% CO_2_. G6PD expression was enhanced by transfecting the cells with a lentiviral vector (CMV enhancer-MCS-polyA-EF1A-zsGreen-sv40-puromycin, Shanghai Gene Chem Co., Ltd., Shanghai, China).

### Western blot, colony formation, and Transwell assays

Total proteins from the HepG2 cells were extracted using phenylmethylsulfonyl fluoride (PMSF)-supplemented radioimmunoprecipitation assay (RIPA) buffer. The protein concentration was determined by using a bicinchoninic acid (BCA) assay kit (Servicebio, Wuhan, China). The protein samples were incubated with anti-G6PD (Affinity Biosciences, Cat# DF6444) and anti-GAPDH (Affinity Biosciences, Cat# AF7021). The HepG2 cell colony formation and Transwell assays were performed as described previously [22].

### SA-β-galactosidase detection assay

The activity of SA-β-gal with HepG2 cell was performed in accordance with the manufacturer guidelines (C0602, Beyotime Biotechnology, Shanghai, China). The stained cells were visualized under an inverted microscope.

### Statistical analysis

Beyond the specified bioinformatics approaches, the data were analyzed using R version 4.1.0 (www.r-project.org) and GraphPad Prism version 8.0. For comparisons between different groups, paired or unpaired *t*-tests were used for normally distributed variables; otherwise, Wilcoxon signed-rank test was used. The relationship between two continuous variables was assessed with Spearman’s rank correlation. A p-value < 0.05 denoted statistical significance.

## Results

### Quality control and annotation of single-cell data

S1 Fig presents the basic information of all individual single-cell samples and the specific details before and after quality control. In total, the screening yielded 33,117 cells. Subsequently, these cells underwent t-SNE dimensionality reduction analysis via the RunTSNE function. The 8 cell subclusters were annotated using classic immune cell markers (S2 Fig).

**Fig 1:**
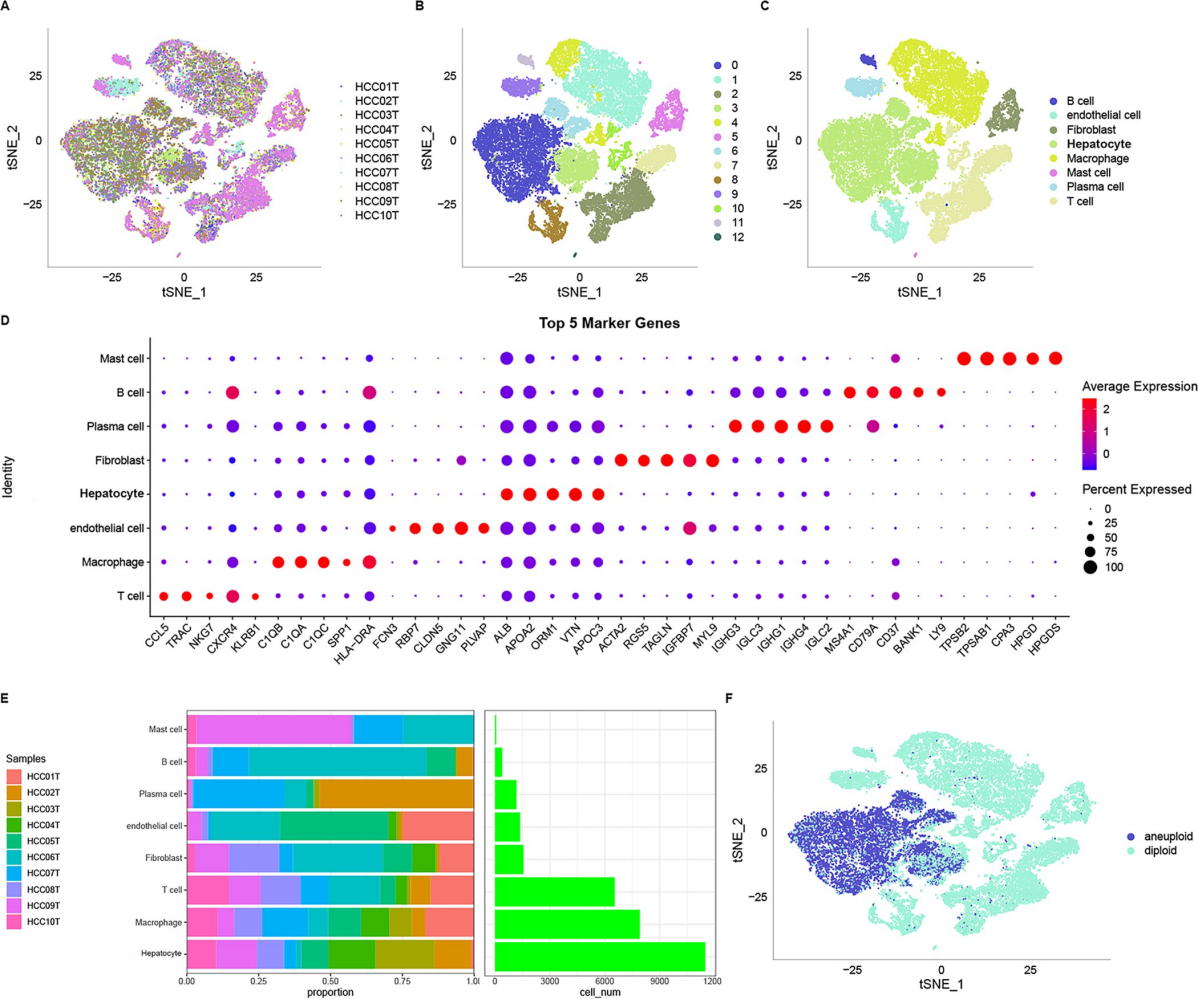
Visualization and annotation of cell subgroups. t-SNE plot of sample distribution (A), clustered subgroups (B), and annotated cell distribution (C). D. Dot plot of marker gene expression in annotated subgroups. E. Proportions and cell numbers of annotated subgroups. F. Malignant and non-malignant cell distribution.

The distribution of 10 samples was illustrated in a t-SNE plot (Fig 1A). Fig 1B showcases the t-SNE plot after clustering, representing the different subclusters. Fig 1C illustrates the distribution of annotated cells in the t-SNE plot. Marker genes for the eight subclusters were identified using the FindAllMarkers function, where genes with an adjusted p-value < 0.05 were selected. The top five most significant marker genes were determined for each subcluster (Fig 1D). Additionally, the proportions of these eight subclusters across all samples were analyzed (Fig 1E). Subsequently, the CNV changes in the single-cell data were predicted using CopyKAT, which enabled the discrimination between tumor cells and normal cells within each sample. Among the cells analyzed, 9,505 were identified as cancer cells, while 20,985 were classified as normal cells. Fig 1F depicts the CopyKAT-generated t-SNE plot, specifically highlighting the predicted malignant cells and normal cells.

### Analysis of aberrant senescence-associated pathways in HCC using bulk RNA-seq and scRNA data

**Fig 2:**
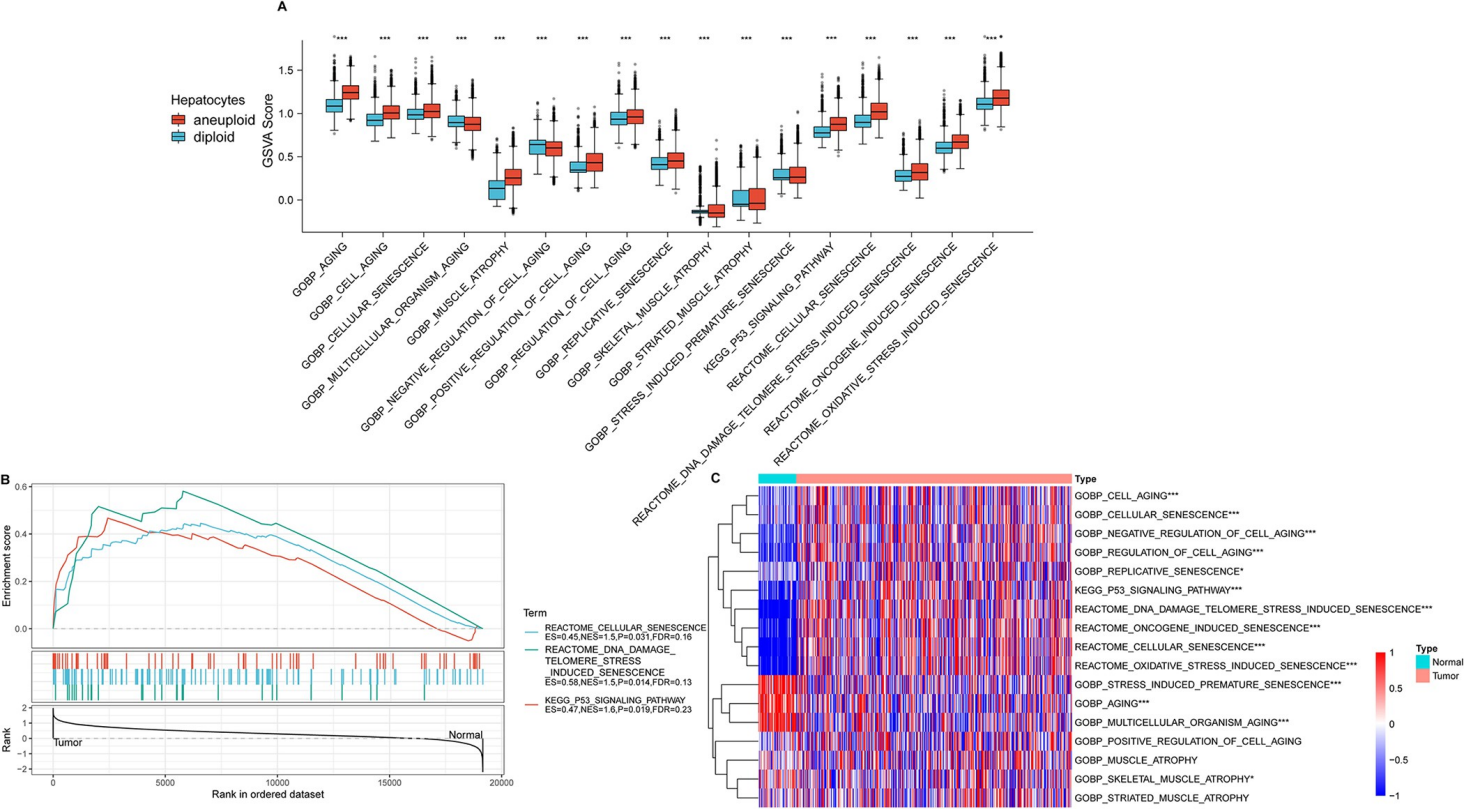
Identification of senescence-related pathways in HCC. A. Single-cell analysis reveals differential senescence-related pathways through gene set variation analysis (GSVA). B. GSEA results for senescence-related pathways in bulk data. C. Comparison of ssGSEA scores for senescence-related pathways in bulk data. *p < 0.05; ***p < 0.001.

Data on the senescence-associated pathways were obtained from the GSEA repository. Subsequently, the respective enrichment scores for these pathways across both malignant and non-malignant cellular contexts were computed using ssGSEA. The scores were standardized by applying z-score normalization based on the enrichment scores for each pathway. There were significant differences in the senescence-related pathway scores between malignant and non-malignant cells (Fig 2A). Following this, the gene expression patterns in 365 tumor and 50 normal tissue samples were analyzed using bulk RNA-seq data from TCGA-LIHC. Initial use of the GSEA software revealed significant enrichments of three pathways exclusively in TCGA dataset tumor tissues: REACTOME_CELLULAR_SENESCENCE, REACTOME_DNA_DAMAGE_TELOMERE_STRESS_INDUCED_SENESCENCE, and KEGG_P53_SIGNALING_PATHWAY (Fig 2B).

Subsequently, the scores of these TCGA-LIHC senescence-related pathways were compared using ssGSEA to evaluate the disparities between the cancerous and adjacent non-cancerous tissues. Importantly, the heat map analysis demonstrated higher enrichment scores for the REACTOME_DNA_DAMAGE_TELOMERE_STRESS_INDUCED_SENESCENCE, REACTOME_CELLULAR_SENESCENCE, and KEGG_P53_SIGNALING_PATHWAY pathways in tumor tissues as compared to the adjacent non-cancerous tissues (Fig 2C). The enriched scores presented compelling evidence for the abnormal activation of the P53 signaling, DNA damage-induced telomere stress-induced senescence, and CS in HCC pathways.

### Construction of senescence-related subtypes with HCC molecular features

**Fig 3:**
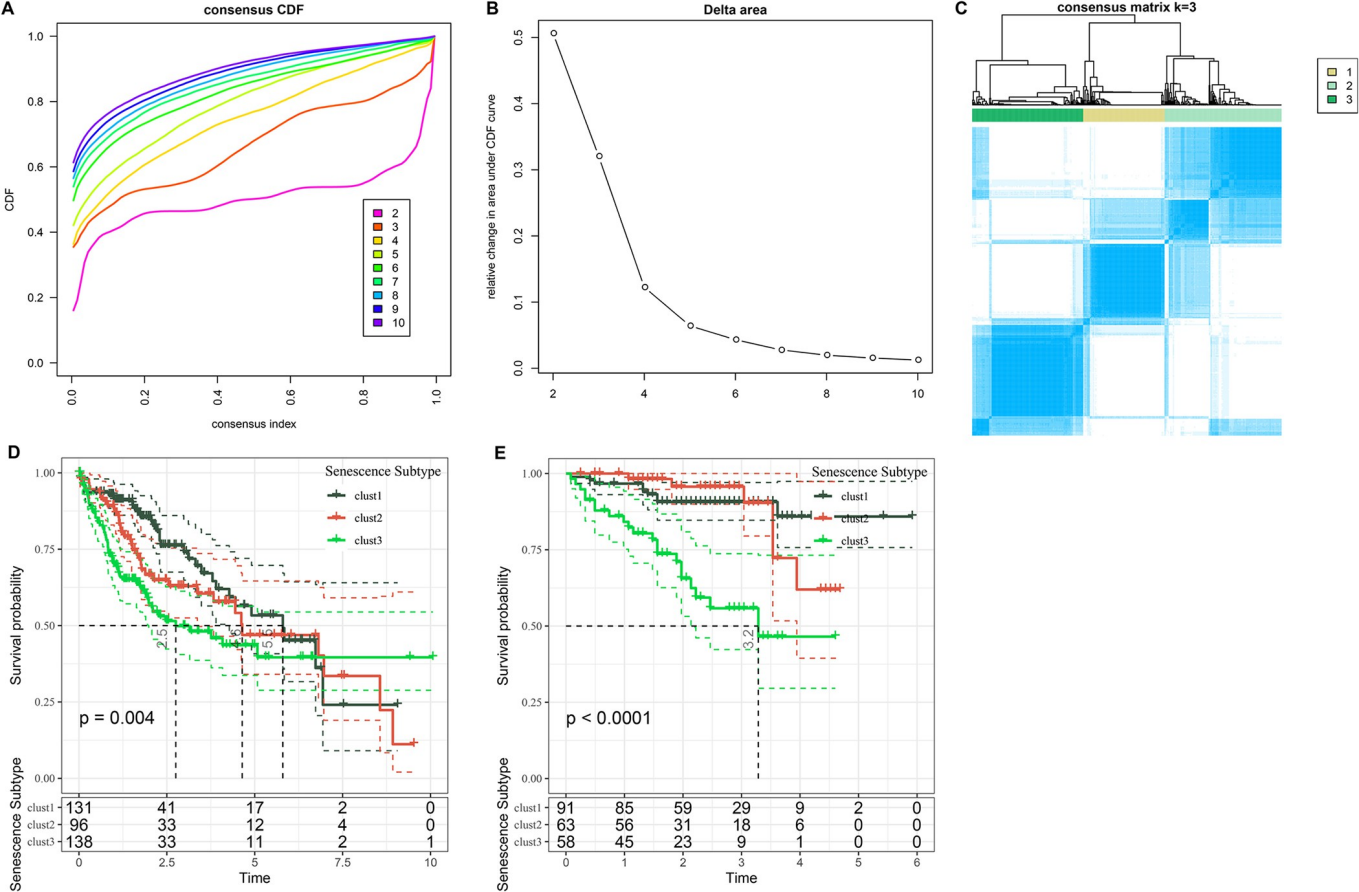
Analysis of subtypes and prognostic implications in HCC cohorts. A. CDF curve for LIHC cohort. B. Change in area curve from consensus clustering analysis. C. Visualization of sample groupings at k = 3 through a heatmap. D. KM survival curve demonstrating the outcomes of three distinct subtypes within TCGA-LIHC cohort. E. KM survival curve illustrating the prognosis of three subtypes in the HCCDB18 cohort.

The earlier analysis highlighted the notable enrichment of the senescence-related P53 signaling, DNA damage-induced telomere stress-induced senescence, and CS in HCC pathways. The gene expression profiles of 186 selected genes within these pathways were consistently clustered, categorizing 365 HCC samples from the TCGA dataset into three distinct clusters. Notably, Cluster 3 demonstrated stable results, as evidenced by the CDF (Fig 3A–3C). Subsequent examination of the prognostic features of these clusters revealed significant disparities in survival outcomes (Fig 3D). Specifically, Cluster 1 exhibited the most favorable prognosis, Cluster 2 was intermediate, and Cluster 3 demonstrated the least favorable prognosis. When this clustering method was applied to HCCDB18 patient data, the prognostic differences observed among the three molecular subtypes (Fig 3E) aligned with TCGA dataset findings. This consistency underscored the robustness of distinguishing senescence subtypes based on key HCC features across diverse study groups.

### Clinical characteristics and outcomes of molecular subtypes

**Fig 4:**
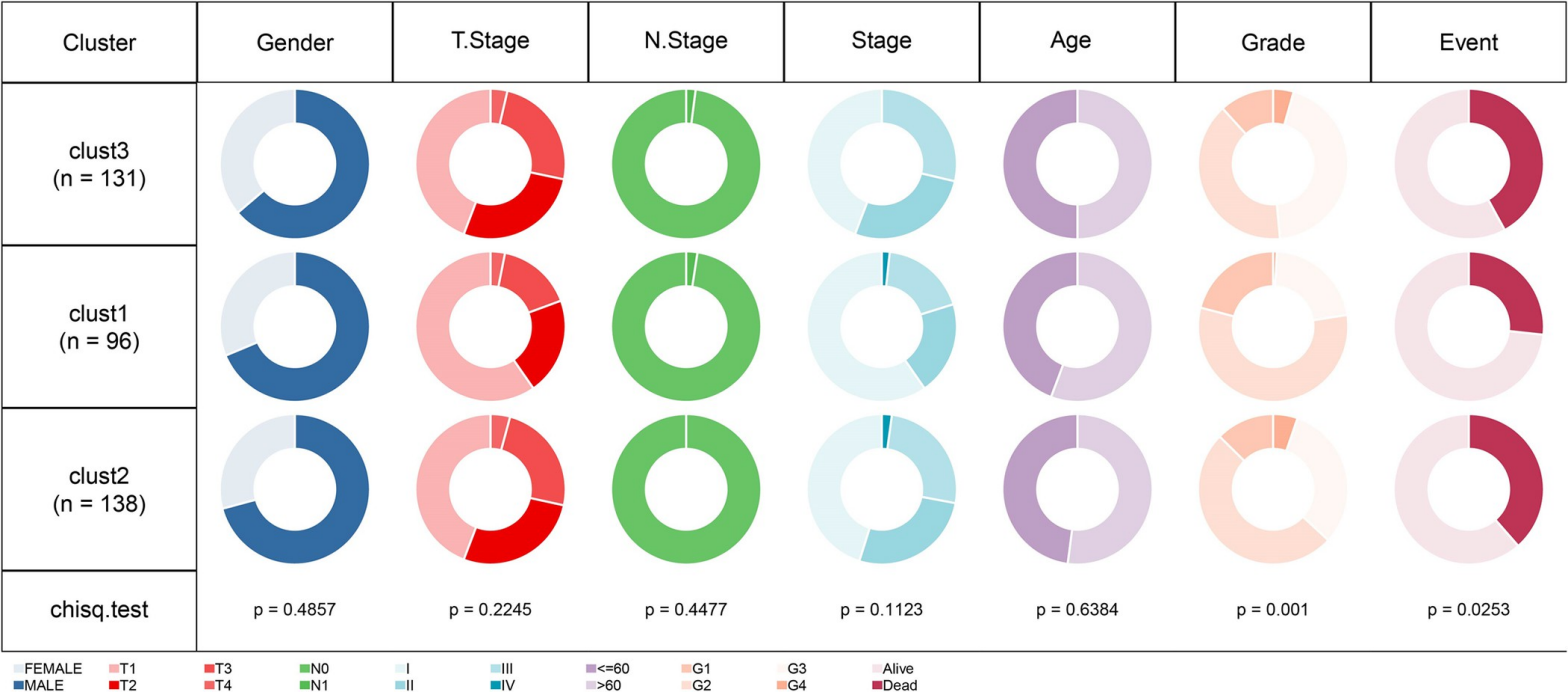
Comparative analysis of clinical feature distribution among molecular subtypes in TCGA-LIHC cohort.

A subsequent exploration of the TCGA-LIHC cohort assessed variations in clinical features among these subtypes. The figure illustrates the distribution of three distinct clusters (clust1, clust2, clust3) across various clinical characteristics, such as gender, tumor stage (including T stage, N stage, and overall stage), age, tumor grade, and survival events. The results of the Chi-square test indicate that the differences in distribution among the clusters for gender, tumor stage (T stage, N stage, overall stage), and age are not statistically significant, with p-values of 0.4857, 0.2245, 0.4477, 0.1123, and 0.6384, respectively. This suggests that these characteristics are relatively balanced across the clusters. In contrast, significant differences are noted in tumor grade (p = 0.001) and survival events (p = 0.0253). The analysis revealed significant disparities in histological grading and clinical outcomes across different senescence subtypes (Fig 4).

The distribution of clinical characteristics among three patient clusters (cluster1, cluster2, cluster3) is illustrated through donut charts, which depict the proportions of various factors including gender, T Stage, N Stage, overall stage, age, tumor grade, and survival events within each cluster. Significant differences were observed in tumor grade (p = 0.001), as well as in survival events (p = 0.0253). Additionally, the number of patients in each cluster is provided in parentheses.

### Biological disparities among senescence subtypes in HCC

**Fig 5:**
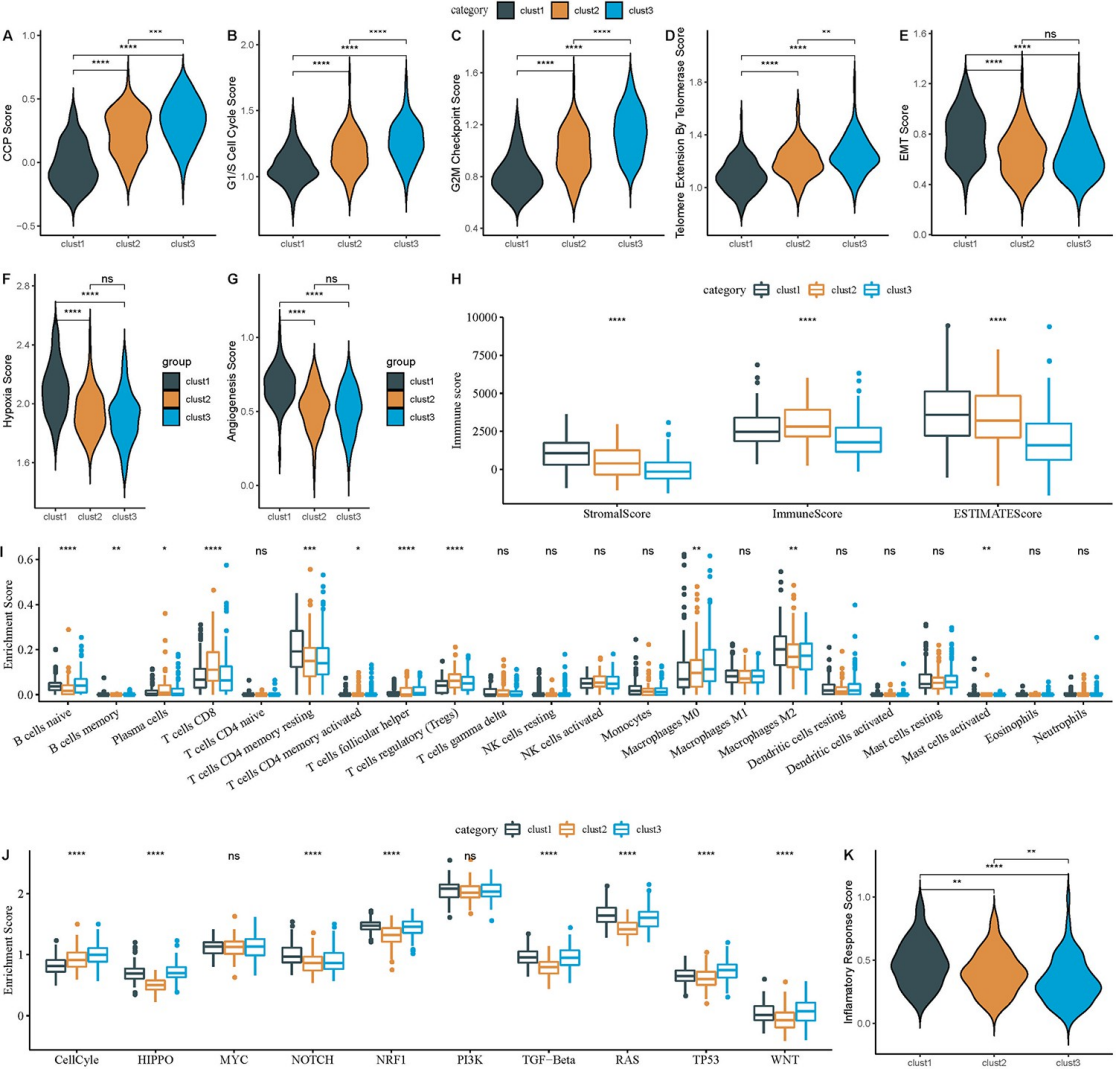
Comparative analysis of scores across three subtypes in TCGA-LIHC dataset. A. CCP scores. B. G1/S phase scores. C. G2M checkpoint scores. D. Telomerase extension scores. E. EMT scores. F. Hypoxia scores. G. Angiogenesis scores. H. Immune scores (ESTIMATE algorithm). I. 22 Immune cell type scores (CIBERSORT algorithm). J. Ten pathway-related tumor scores. K. Inflammatory factor scores. ns, p ≥ 0.05; *p < 0.05; **p < 0.01; ***p < 0.001; ****p < 0.0001.

CS is pivotal in tumor progression through diverse pathways and was investigated by examining the biological disparities among various previously identified senescence subtypes. Prior research posited that cancer cells induce CS by impeding the cell cycle. The scores for the cell cycle process in each sample were computed using 31 cell cycle-associated genes [18], and then the scores were compared among the senescence subtypes. Intriguingly, the Cluster3 subtype, associated with the poorest prognosis, manifested an elevated score in the cell cycle process (Fig 5A). Furthermore, the G1/S phase and G2 checkpoint score assessment revealed that the cluster3 subtype registered high scores for both (Fig 5B and 5C).

Tumor cells tend to activate telomerase activity to mitigate telomere attrition [17]. Consistent with this, the telomere elongation analysis indicated that the cluster3 subtype demonstrated higher scores (Fig 5D). Furthermore, senescent cells might influence tumor migration and metastasis by releasing cytokines that stimulate epithelial–mesenchymal transition (EMT). Contrary to expectations, the cluster3 subtype exhibited a diminished EMT score, suggesting reduced activity (Fig 5E). The exploration of hypoxia and the angiogenesis scores determined that the cluster1 subtype had elevated scores for both (Fig 5F–5G). The immune and stromal scores revealed that the cluster3 subtype had the least immune activity (Fig 5H). Examining the scores for 22 immune cell types highlighted significant variances across the senescence-related subtypes. Specifically, Clusters 2 and 3 are predominantly associated with enhanced infiltration of tumor-suppressive T cells and B cells, demonstrating significant correlations. In contrast, Cluster 1 is associated with tumor-promoting M2 macrophages, also showing significant relevance. However, there is no apparent correlation between natural killer (NK) cells, dendritic cells (DCs), and eosinophils with the senescence-related cluster. (Fig 5I). Furthermore, the evaluation of 10 tumor-related pathways revealed disparities among the subtypes (Fig 5J). Lastly, the inflammatory score assessments revealed that Cluster 3 had significantly lower scores than Cluster 1 and Cluster 2 (Fig 5K). Collectively, the results highlighted the pronounced differences in multiple biological attributes such as cell cycle, telomere elongation, EMT, hypoxia, angiogenesis, immune response, and inflammation across different senescence subtypes. This evidence emphasized the viability of differentiating senescence subtypes in HCC based on molecular markers.

### Differential mutation features among senescence subtypes in HCC

**Fig 6:**
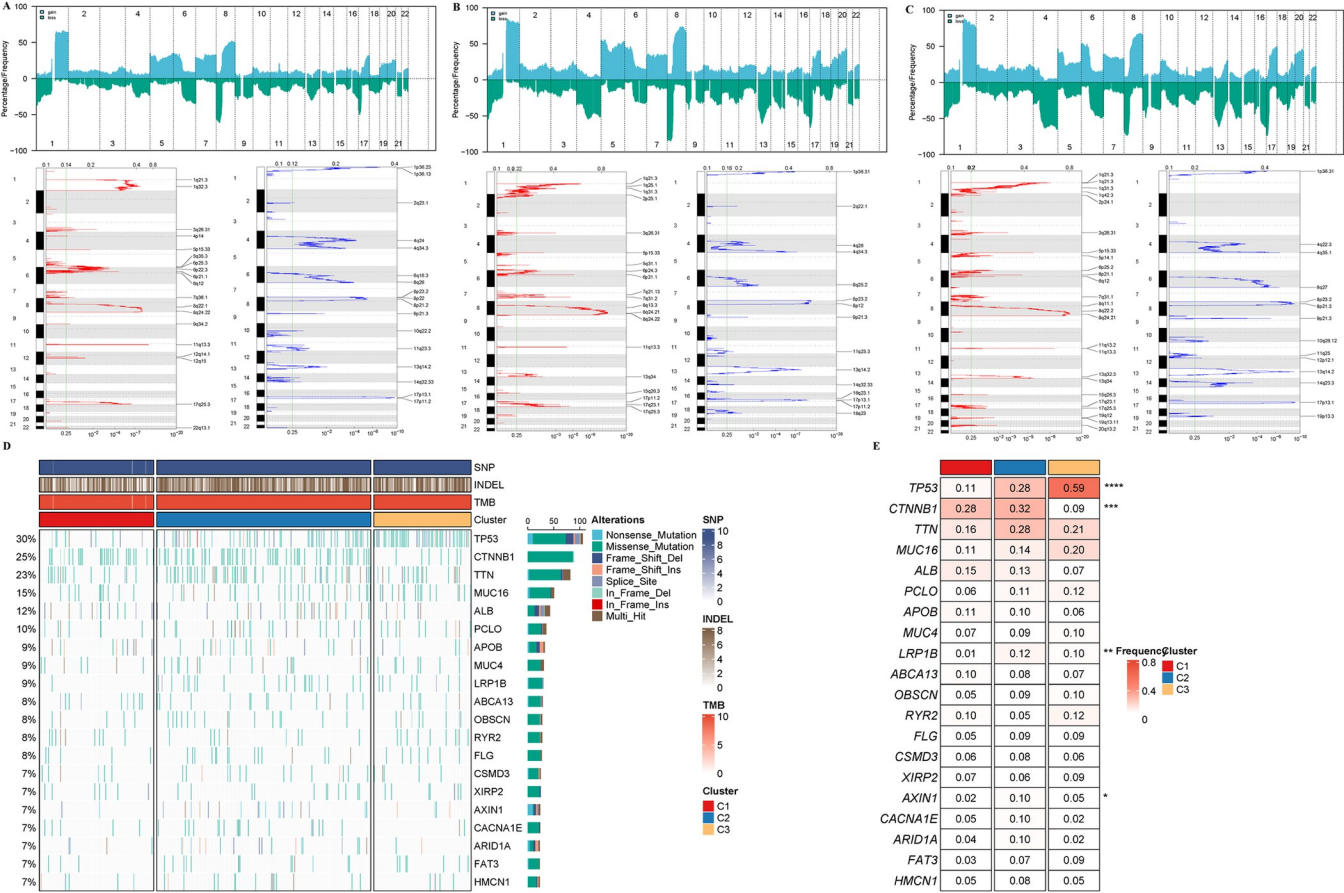
CNV and SNV mutation analysis. Peak plot of CNV in cluster1 subtype (A), cluster2 subtype (B), and cluster3 subtype (C). D. The waterfall plot depicted the differences in frequently mutated genes of HCC among three clusters. The right panel shows the mutation rate, and genes were ordered by their mutation frequencies. E. Mutation frequency of the top 20 frequently mutated genes among three clusters.

The CNV results from TCGA-LIHC were integrated using GISTIC2. During the integration, a confidence level of 0.9 was selected, and the analysis was conducted using hg38 as the reference genome. The analysis results demonstrated notable variances in the CNV among the subtypes (Fig 6A–6C). We further depicted the genomic variation map among the three heterogeneous clusters. As shown in Fig 6D, the mutation frequencies of the top 20 frequently mutated genes are displayed, along with the profiles of SNP, INDEL, and TMB. To enhance the understanding of somatic mutations, we compared the mutation differences of the top 20 frequently mutated genes among the three clusters. There were significant differences in the mutation frequencies of TP53, CTNNB1, LRP1B, and AXIN1 among patients from different clusters (Fig 6E).

### Development of signature derived from senescence-related subtypes in HCC and functional validation of G6PD in HepG2 Cells

**Fig 7:**
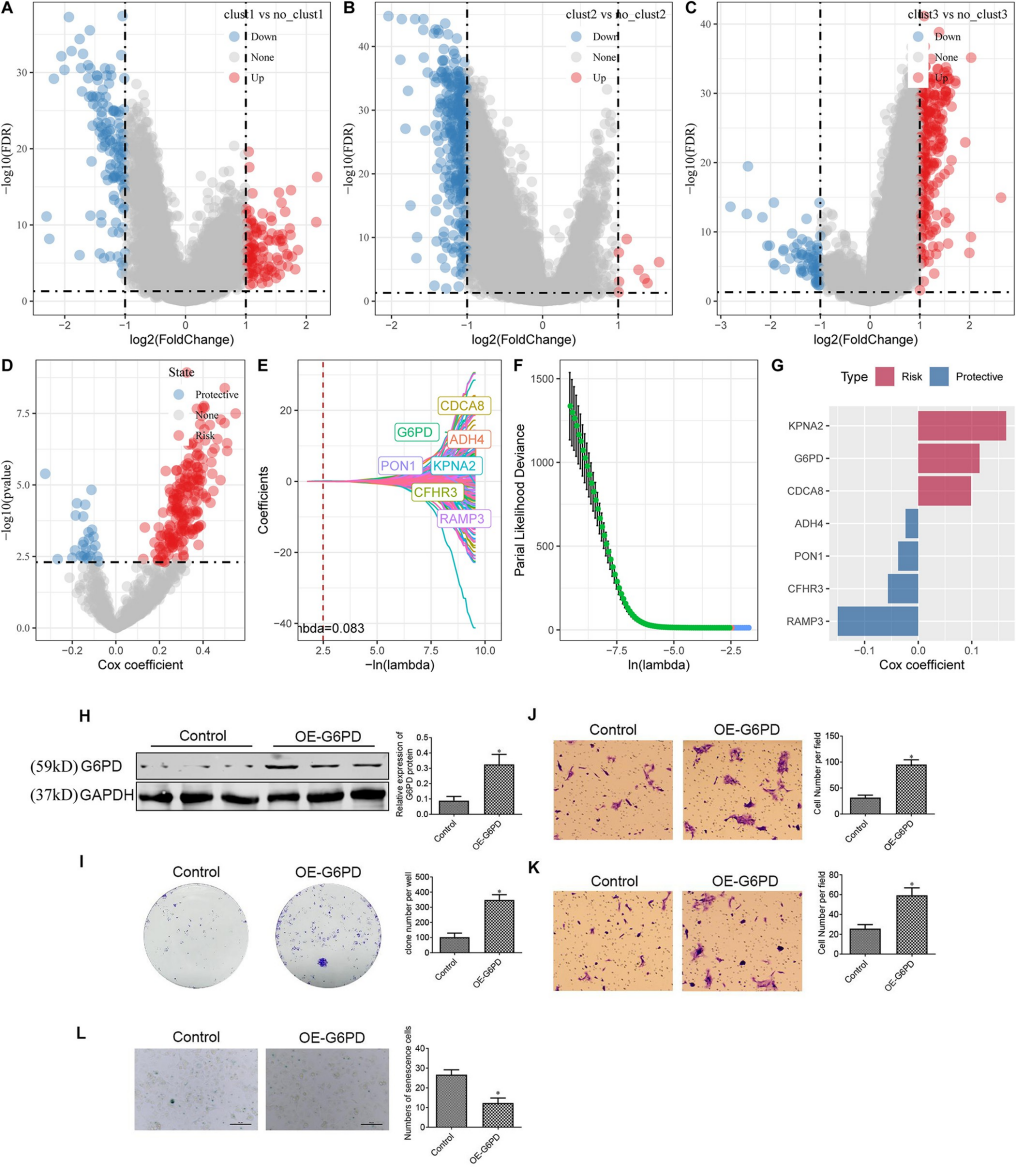
Differential gene screening among three subtypes and risk model construction. Volcano plot for differential analysis of cluster1 vs. no_cluster1 (A), cluster2 vs. no_cluster2 (B), and cluster3 vs. no_cluster3 (C). D. Identification of 729 candidates among differentially expressed genes. E. Trajectory of each independent variable as lambda changes. F. Confidence interval under lambda. G. Coefficients of the seven genes in the model. H. Western blot assay to evaluate G6PD protein expression in HepG2 cells. I. Colony formation assay to evaluate HepG2 cell proliferation. J-K. Transwell assay assesses HepG2 cell migration (J) and invasion (K). L. SA-β-gal staining of HepG2 cell. *p < 0.05.

The earlier analysis aimed to identify three distinct molecular subtypes based on genes related to HCC-specific senescence pathways. The results revealed biological characteristics and clinical phenotype differences between these subtypes. Specifically, Cluster 3 exhibited the poorest prognosis, followed by Cluster 2, while Cluster 1 had the most favorable prognosis. Subsequently, differential analysis for Cluster 1 vs. non-Cluster 1 subtypes, Cluster 2 vs. non-Cluster 2 subtypes, and Cluster 3 vs. non-Cluster 3 subtypes was conducted on the 365 HCC samples from the TCGA-LIHC dataset using the limma package. Genes demonstrating significant differences were identified based on p < 0.05 and |log2 (FC) | > 1. Thus, there were 111 upregulated genes and 127 downregulated genes in Cluster 1 vs. non-Cluster 1, eight upregulated genes and 357 downregulated genes in Cluster 2 vs. non-Cluster 2, and 245 upregulated genes and 68 downregulated genes in Cluster 3 vs. non-Cluster 3. In total, 729 differentially expressed genes were selected for further analysis. The differential analysis results for Cluster 1 vs. non-Cluster 1, Cluster 2 vs. non-Cluster 2, and Cluster 3 vs. non-Cluster 3 are illustrated in volcano plots (Fig 7A–7C).

The influence exerted by the 729 differentially expressed genes was examined using univariate Cox regression analysis. The results revealed that 220 genes (194 “risk” genes and 26 “protective” genes) significantly influence TCGA-LIHC cohort prognosis (p < 0.005) (Fig 7D).

The risk model was refined using LASSO regression, specifically using the R glmnet package for LASSO Cox regression. The trajectory of each independent variable was assessed as lambda increased (Fig 7E). As lambda values increased, the number of coefficients tending towards zero gradually increased. The model was constructed using 10-fold cross-validation, and the confidence intervals were examined for each lambda value (Fig 7F). The results indicated that the optimal model was achieved when lambda was set to 0.0830. Subsequently, seven genes corresponding to lambda = 0.0830 were selected as the target genes for subsequent analysis (Fig 7G). The final seven-gene signature formula is presented below:

RiskScore=0.099×CDCA8+0.114×G6PD—0.024×ADH4–0.057×CFHR3+0.164×KPNA2–0.15×RAMP3–0.037×PON1

The role of G6PD protein in HepG2 cells was examined. Western blotting (Fig 7H) demonstrated enhanced G6PD protein expression in the OE(Overexpression)-G6PD group as compared to the control group, thus confirming successful transfection. Cell proliferation was assessed using the colony formation assay (Fig 7I), which revealed significant growth effects by the OE-G6PD group on HepG2 cells as compared to the control group. Furthermore, the Transwell assay (Fig 7J–7K) revealed notable augmentation of the HepG2 cell migration and invasive capacities consequent to the G6PD overexpression. The results were similar to those observed in SK-HEP-1 cell (S3 Fig). We also stained for senescence-associated β-galactosidase (SA-β-Gal), a commonly accepted marker for senescent cells. The number of SA-β-Gal HepG2 cell was decreased in Si- G6PD group compared to control (Fig 7L), suggesting that overexpression of G6PD suppress cellular senescence of HepG2 cell.

### Application and validation of senescence-derived signature in HCC

**Fig 8:**
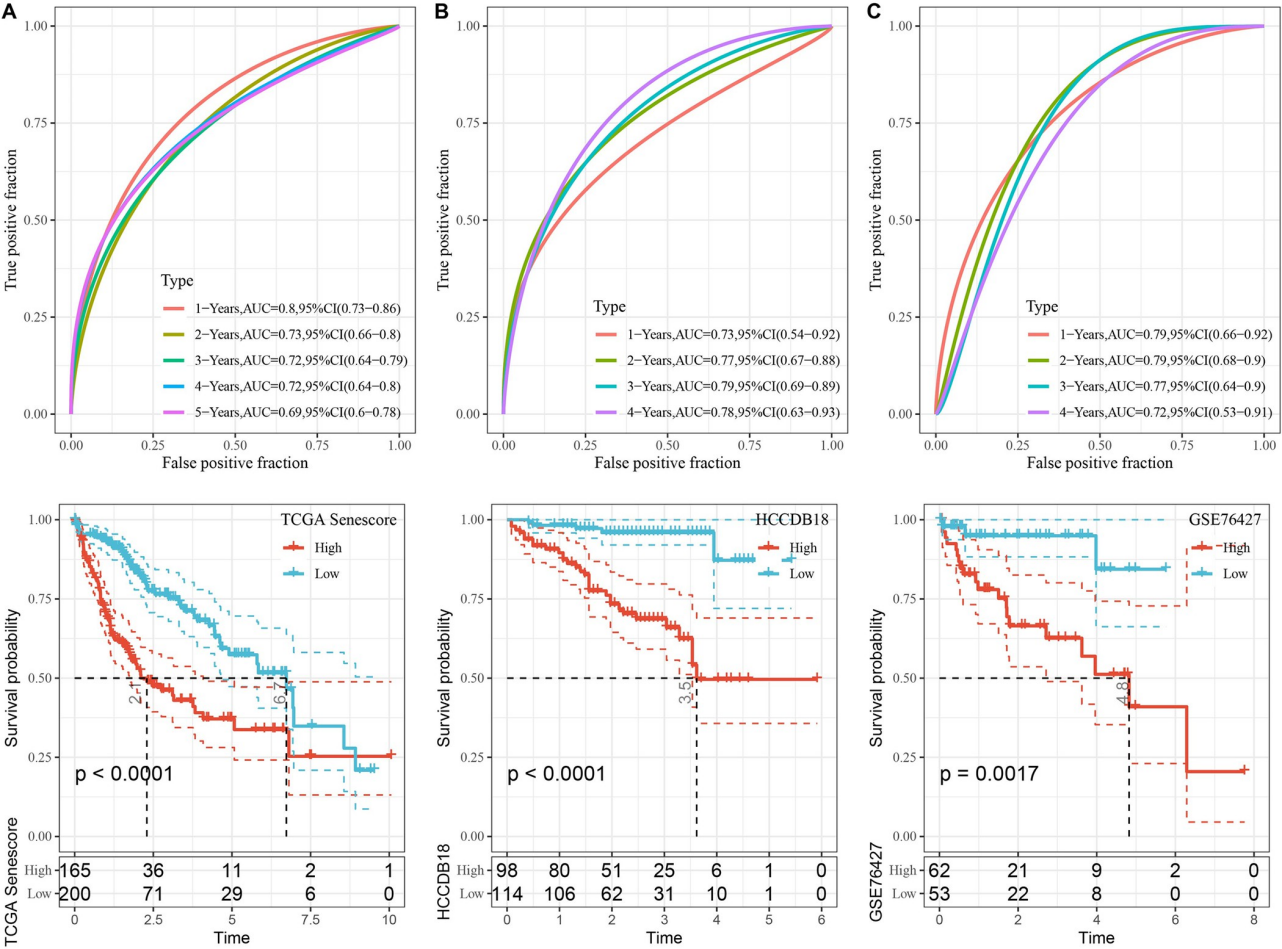
Performance evaluation of seven-gene risk models from TCGA, HCCDB18, and GSE76427 datasets. A–C. ROC curves and KM curves of the risk model constructed from seven TCGA dataset genes (A), seven HCCDB18 dataset genes (B), and seven GSE76427 dataset genes (C).

The expression profiles of seven predetermined genes were evaluated across various samples using TCGA-LIHC dataset as the training cohort. Subsequently, a corresponding RiskScore was calculated for each sample. These RiskScores underwent ROC analyses via the R timeROC package to evaluate their prognosis classification. The prognostic prediction efficiency was distinctly evaluated for year-long intervals of up to five years. Remarkably, the derived model exhibited substantial efficacy, as indicated by a notable AUC in the ROC analysis. Subsequently, the RiskScores were normalized and converted into z-scores. Samples presenting z-scores > 0 were categorized as high-risk, whereas those with z-scores < 0 were designated as low-risk. The Kaplan-Meier (KM) survival curves revealed a statistically significant disparity in the prognostic outcomes between the high- and low-risk groups (Fig 8A).

The methodology was validated via application to two independent datasets: HCCDB18 and GSE76427. The findings corroborated the findings that the seven-gene risk model demonstrated remarkable consistency and robustness across varying cohorts. Notably, a significant divergence in prognostic outcomes was evident between the high- and low-risk classifications in both the HCCDB18 and GSE76427 datasets (Fig 8B and 8C).

### Association between senescence-derived RiskScore and HCC clinical and biological features

**Fig 9:**
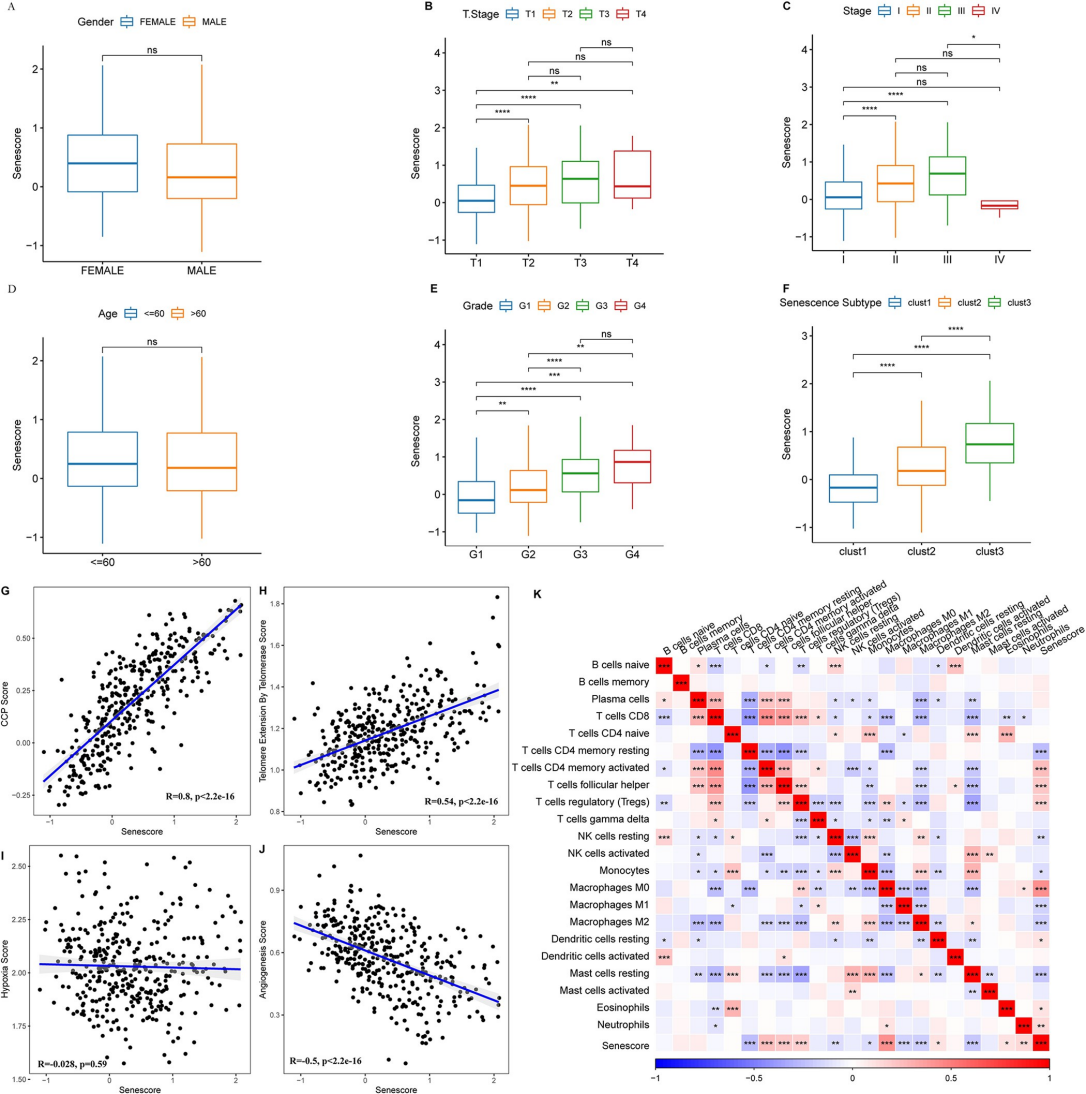
Association between RiskScore and clinical phenotypes and biomarkers. A–F. Differences in risk scores across clinical phenotypes. Scatter plot demonstrating the correlation between CCP scores and RiskScore (G), between telomerase telomere extension scores and RiskScore (H), between hypoxia scores and RiskScore (I), and between angiogenesis scores and RiskScore (J). K. Heatmap depicting the correlation between immune cell infiltration predicted by CIBERSORT and RiskScore. ns, p ≥ 0.05; p < 0.05; **p < 0.01; ***p < 0.001; ****p < 0.0001.

The RiskScore was comprehensively analyzed across diverse clinical phenotypes in TCGA-LIHC dataset to delineate the association between the RiskScore and HCC clinical attributes. The data suggested a direct correlation between higher clinical grade and a concomitant rise in RiskScore (Fig 9A–9F). Previous investigations highlighted connections between senescence-associated subtypes and various cellular processes, namely cell cycling, telomerase functionality, hypoxic response, angiogenesis, and immune response. The interrelation between these feature scores and the senescence-associated RiskScore was identified. The investigation revealed a marked positive correlation between the CS score and the CCP score (Fig 9G). Simultaneously, a pronounced positive linkage between the CS and telomerase elongation scores was recorded, juxtaposed with a negative association with angiogenic activity (Fig 9H–9J). Additionally, there was a significant association between immune cell infiltration levels and the senescence score (Fig 9K).

### Role of senescence-derived RiskScore in immunotherapy and chemotherapy

**Fig 10:**
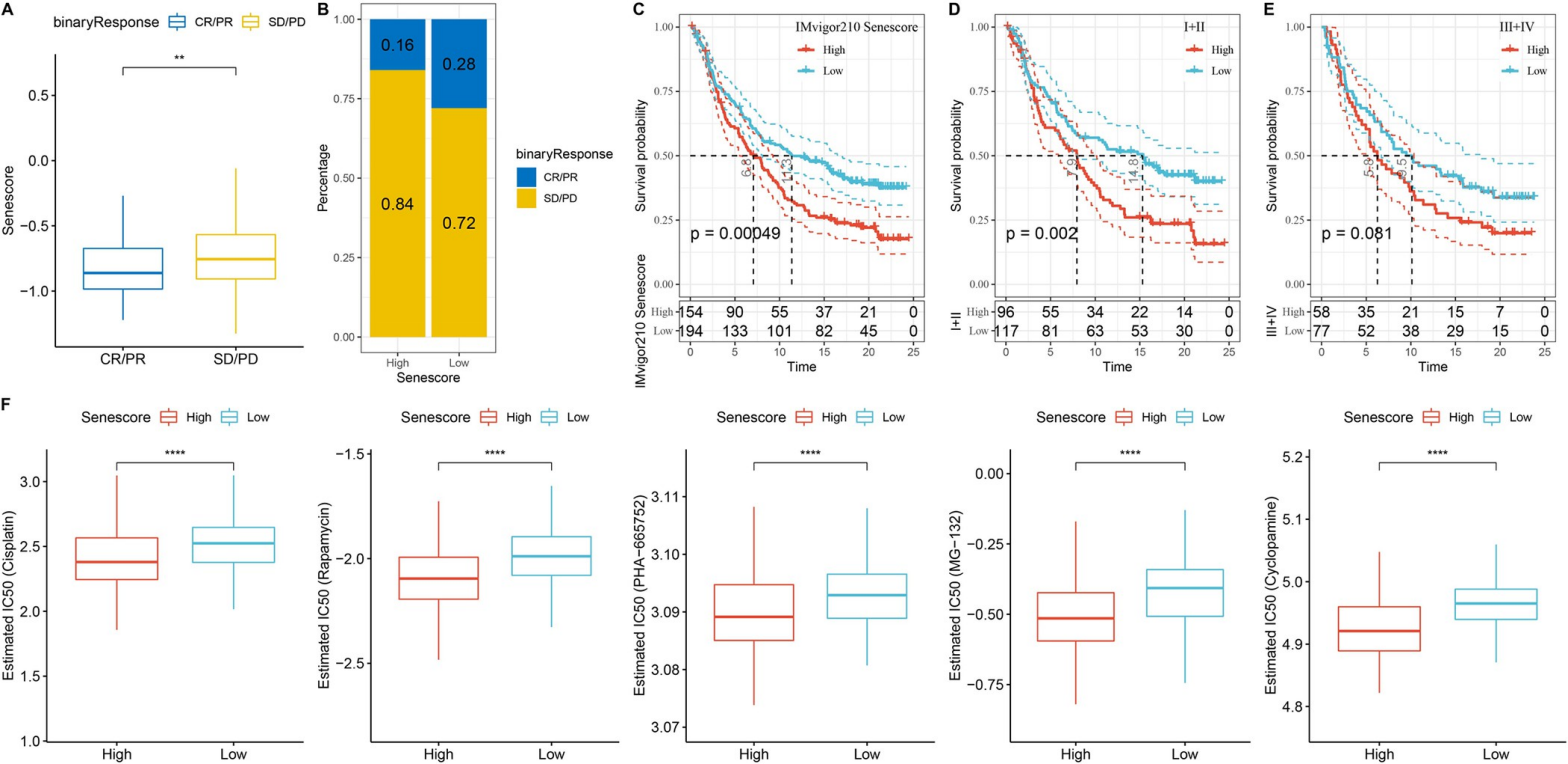
Exploring RiskScore and its influence on immunotherapy and drug sensitivity. A. Differential RiskScore among immunotherapy responses. B. Distribution of immunotherapy response among RiskScore groups. C. Prognostic differences among RiskScore groups. Prognostic differences among RiskScore groups in early-stage patients (D) and advanced-stage patients (E). F. Differential drug sensitivity between high and low RiskScore groups in TCGA-LIHC cohort. **p < 0.01; ****p < 0.0001.

In this exploration of the association between RiskScore and immunotherapy efficacy, the capacity of RiskScore to prognosticate patient outcomes following immune checkpoint blockade (ICB) therapy was examined, with emphasis on the anti-PD-L1 cohort (IMvigor210) [23]. This cohort comprised 348 people who exhibited a spectrum of responses to PD-L1 receptor blockade that spanned partial response (PR), complete response (CR), progressive disease (PD), and stable disease (SD). Notably, individuals in the SD/PD response group had elevated RiskScore levels compared to their counterparts exhibiting other types of responses (Fig 10A).

The percentage analysis between the low and high RiskScore groups revealed significantly better treatment response in the low RiskScore group (Fig 10B). The findings indicated that patients with higher RiskScore had poorer outcomes following treatment (p < 0.05) (Fig 10C). The survival differences among all samples in the IMvigor210 cohort and within different stages were explored. The results demonstrated significant survival disparities in Stage I+II samples (Fig 10D). Contrastingly, no significant survival differences were observed between the high and low RiskScore groups in Stage III+IV samples (Fig 10E). Particularly noteworthy is the exceptional predictive performance of the RiskScore in early-stage clinical samples. Additionally, the response level to traditional chemotherapy drugs, such as cisplatin, was analyzed in the high- and low-risk groups. The results revealed that the high RiskScore group had higher sensitivity to these drugs (Fig 10F).

### Clinical implications of the senescence-derived RiskScore in HCC

**Fig 11:**
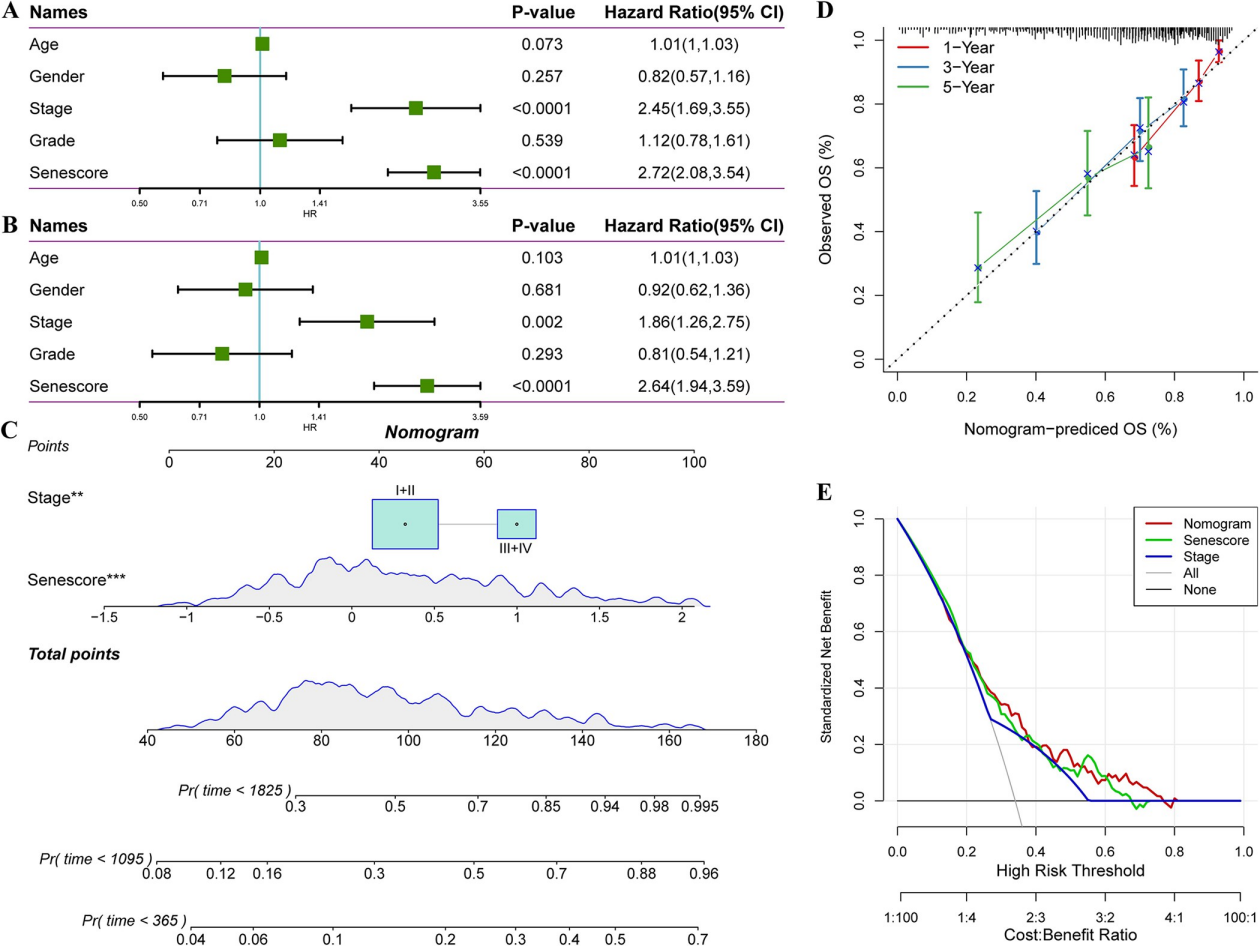
Statistical model analysis of RiskScore and clinical clinicopathological features. Univariate (A) and multivariate (B) Cox analyses of RiskScore and clinical pathological features. C. Nomogram model. D. Calibration curves of the nomogram at one, three, and five years. E. Nomogram decision curve. **p < 0.01; ***p < 0.001.

The univariate and multivariate Cox regression analyses of the RiskScore and the HCC clinicopathological features revealed that the RiskScore exerted the most significant prognostic effect (Fig 11A and 11B). The RiskScore was combined with other clinicopathological features to quantify patient risk assessment and survival probability, and a forest plot was constructed (Fig 11C). The model results indicated that the RiskScore had the greatest influence on survival prediction. Furthermore, the predictive accuracy of the model was assessed using calibration curves (Fig 11D). The calibration curves for the one-, three-, and five-year time points closely aligned with the standard curve, suggesting the excellent predictive performance of the forest plot. Moreover, the reliability of the model was evaluated using decision curve analysis (DCA) (Fig 11E). Both the RiskScore and nomogram demonstrated substantial net benefits compared to the extreme curve. Compared to other clinicopathological features, the nomogram and RiskScore exhibited the strongest survival prediction ability.

## Discussion

HCC represents a highly heterogeneous malignancy characterized by diverse pathogenic determinants and an increased incidence rate [24]. Contemporary postoperative outcomes for patients with HCC remain less than optimal. A significant proportion of patients with HCC exhibit reduced susceptibility to multi-target receptor tyrosine kinase inhibitors and immune checkpoint inhibitors (ICIs), which are exemplified by PD-1/PD-L1 antagonists [25]. This proportion underscores the importance of precisely identifying a prognostic biomarker to inform therapeutic decisions [26]. A pivotal cellular phenomenon in senescence and tumorigenesis in HCC, CS warrants elucidation, specifically regarding the molecular characteristics of senescence, immune profiles, clinical manifestations, and treatment responsiveness.

Based on the above, a scoring metric predicated on mRNA expression landscapes in HCC was devised in the present study. The metric facilitated correlations between senescent molecular subtypes and the genomic, immune, and clinical spectra in patients with HCC. Significantly, risk stratification using CS features proficiently predicted patient outcomes and revealed directions for therapy. Collectively, the findings present a comprehensive paradigm to understand the CS-associated markers in HCC, catalyzing further investigations and biomarker discovery.

The present study identified the HCC-specific senescence-associated pathways. While numerous senescence-centric pathways demonstrated differential significance, only three signaling pathways were significant following integrated single-cell data and GSEA screening. Earlier investigations explored the senescence hallmark pathways in clear cell renal cell carcinoma [27]. However, the defined pathways markedly differed from those in the present study, which highlighted the distinctive molecular nuances of senescence in dissimilar tumors [28]. Concurrent studies on prostate cancer examined its molecular heterogeneity and immune milieu dynamism, where three CS genes with prognostic potential were isolated [29]. Aligning with these insights, the present study also constructed a multi-gene model for HCC as a prospective tool for predicting prognoses and evaluating therapeutic responses. The results suggested the intrinsic flexibility of senescence attributes across tumor types, likely modulated by the inherent traits of the tumor and the surrounding immune environment. A profound understanding of senescence attributes can inspire a more refined understanding of senescence mechanisms across diverse malignancies, thereby refining prognostic evaluations and tailoring therapeutic interventions for HCC.

The molecular biological variances across diverse senescence subgroups were investigated, where distinctions in signaling pathways and variations in tumor characteristic expression were emphasized. Numerous senescence features mirror those of cancer. However, their interplay in tumorigenesis remains debated. The common hallmarks shared by senescence and cancer include genomic instability, epigenetic alterations, persistent inflammation, and ecological dysregulation, collectively referred to as “meta-features”. Despite lacking direct counterparts in cancer, certain senescence features (impaired protein homeostasis, mitochondrial dysfunction, and altered intercellular communication) are also considered detrimental factors that contribute to tumor progression. Interestingly, other specific senescence features, such as telomere attrition and stem cell exhaustion, counteract the emergence of cancer hallmarks, including infinite replicative potential and phenotypic versatility, thereby acting as antagonistic attributes.

This study highlights the potential biological traits of the three clusters, with Cluster 3 (C3), linked to the poorest prognosis, being of particular interest. Previous research has established a strong link between TP53 mutations and an increased tumor mutation burden, as well as poorer outcomes in patients with HCC [30]. Our findings corroborate this association within the C3 subtype, revealing that TP53 mutations occur at the highest frequency in this group, aligning with the trend of poorer patient outcomes noted in existing literature. In a noteworthy development, previous studies have established a significant correlation between Wnt/CTNNB1 mutations and resistance to immunotherapy, suggesting that these mutations are associated with a poor prognosis [31]. However, our research reveals that the frequency of CTNNB1 mutations within the C3 subtype is, in fact, the lowest among all subtypes. This unexpected result raises questions about the biological implications of CTNNB1 mutations, suggesting they may exert different effects depending on the molecular context or that their prognostic relevance in certain subtypes could be influenced by other overriding factors. Furthermore, the biological characteristic scores across the subtypes reveal that patients in the C3 subtype exhibit elevated scores for both angiogenesis and cell cycle activity, indicating a more aggressive tumor behavior. However, Cluster 3 had lower levels of other characteristics, such as EMT and vascular formation. Interestingly, although these results appear contradictory, similar patterns were identified when analyzing the senescence subtypes in other tumors [27]. Moreover, two other senescence characteristics have been highlighted: disabled macroautophagy and CS, whose tumor-suppressive and tumor-promoting roles are environment-dependent [32]. Therefore, it is suggested that senescence does not always promote the malignancy process in tumors. By selectively inhibiting the detrimental functions of senescence and amplifying the beneficial effects of CS, it might be possible to offset the adverse factors associated with senescence in tumor treatment. Thus, the heterogeneity in malignant attributes across senescence subgroups highlights the potential of stratifying HCC based on senescence phenotypes.

Senescence-associated molecular characteristics were systematically quantified in HCC to define their clinical implications. The resultant quantified attributes were then encapsulated into a risk score metric. The primary focus was to discern correlations between risk scores extrapolated from these senescence molecular markers and pivotal clinical indices: patient prognosis, intricate pathological nuances, and oncological pharmacotherapy responsiveness. A cross-sectional data analysis underscored discernible variations in the senescence risk score when compared across a spectrum of pathological gradations and histopathological echelons. Remarkably, the prognostic algorithm demonstrated substantial precision in predicting the clinical course of patients with HCC. Furthermore, the risk score established its position as a standalone prognostic biomarker, a claim substantiated across diverse HCC cohorts, thereby cementing the robustness of the investigative outcomes.

Notably, in clinical practice, the judicious selection of therapeutic agents is paramount for overcoming drug resistance, augmenting objective response rates, and enhancing overall survival, particularly in patients with advanced HCC. Phase III trials that examined sorafenib reported that the ORR defined by the modified Response Evaluation Criteria in Solid Tumors (mRECIST) was 10%–15% [33]. Contrastingly, lenvatinib-treated patients had an ORR of 24.1% [34]. In this context, the potential of a senescence risk score to prognosticate drug efficacies in HCC cohorts was evaluated. The analysis results highlighted that patients in the high-risk scores group had heightened susceptibility to established chemotherapeutic agents, exemplified by cisplatin. Developing the senescence risk quantifier could potentially revolutionize therapeutic stratification, particularly for patients in advanced disease stages or with bleak prognostic outlooks, thereby facilitating enhanced clinical resolutions.

The applications of the senescence risk score also extend to immunotherapy. Notably, the CheckMate-040 trial [1], which spotlighted nivolumab as a monotherapeutic agent, heralded a new era in HCC immunotherapy. The idea was echoed by the subsequent KEYNOTE-224 study that underscored the therapeutic ability of pembrolizumab in the HCC therapeutic landscape [35]. Despite the advances in ICIs, monotherapeutic ICI paradigms, particularly those using PD-1/PD-L1, have not achieved their envisioned therapeutic peak. Hence, augmenting ICI therapeutic outcomes through novel regimens remains imperative. The preliminary insights of the present study highlight the prognostic potential of the risk score in immunotherapeutic cohorts. Alarmingly, patients categorized as high-risk had a suboptimal clinical prognosis post-immunotherapy. This finding suggested the necessity of an exhaustive clinical appraisal of this cohort to delineate optimal therapeutic strategies. Based on the abovementioned findings and discussions, it is cautiously posited that formulating a senescence risk scoring system is promising for augmenting the efficacy of existing therapies for patients with HCC.

In this study, we have confirmed the significance of G6PD as a biomarker for HCC within the framework of CS. G6PD is essential for providing the reducing power necessary for cellular growth and for maintaining redox balance [36]. Recent research has shown that G6PD is involved in various cellular processes beyond just red blood cell diseases, particularly through redox signaling. When G6PD activity is lacking, it can weaken antioxidant defenses and disrupt cell signaling pathways. Moreover, abnormal activation of G6PD has been associated with the initiation and progression of tumors. Previous studies have highlighted the critical roles of G6PD in regulating apoptosis, as well as its involvement in STAT3- and STAT5-mediated tumorigenesis, colon cancer metastasis, and cancers with p53 mutations [37,38]. We propose that G6PD affects cellular senescence in HCC through several regulatory pathways. Firstly, G6PD is vital for maintaining redox balance, which is crucial for the proper functioning of antioxidant defense mechanisms, thereby minimizing oxidative stress accumulation during cellular senescence. Secondly, G6PD is implicated in the regulation of key signaling pathways, such as STAT3 and STAT5, which are known to be significant in both senescence and tumorigenesis. Additionally, aberrant G6PD activation may inhibit apoptosis, resulting in the accumulation of damaged cells and worsening cellular senescence. Overall, G6PD may regulate the senescence process in HCC cells through multiple pathways, influencing tumor progression and malignancy. However, the precise regulatory mechanisms of G6PD in this context require further investigation and validation in future studies.

Preliminary data indicate a connection between the CS score and clinical outcomes, as well as sensitivity to immunotherapy and chemotherapy in HCC. However, it is essential to acknowledge the limitations of this study. Firstly, we define senescence characteristics in HCC patients using transcriptomic information. However, with the advancement of omics technologies, the integration of additional omics data, such as proteomics and metabolomics, has the potential to substantially enhance our comprehension of senescence characteristics across diverse patient classifications. Secondly, although the predictive ability of the risk model has been validated in other datasets, the variability among individual patients and the diversity of treatment methods may hinder its broader clinical application. Therefore, extensive validation in real-world settings remains a critical future research objective. Moreover, we selected the gene G6PD as a preliminary marker in our analysis, which may result in the oversight of other important genes and limit the comprehensiveness of the model. Consequently, further exploration of additional genes included in the model is warranted. Lastly, the cohorts currently involved in immunotherapy studies may not be derived from HCC populations, potentially affecting the effectiveness of evaluating the CS score in relation to immunotherapy. These limitations highlight the need for further enhancements in future research.

## Conclusions

In summary, the study investigated hepatocellular carcinoma (HCC)-specific senescence-related pathways and developed two prognostic models utilizing genes associated with these pathways. The risk model, which characterizes senescence in HCC, demonstrated robust predictive efficacy. Notably, the risk score effectively guided clinical outcomes for patients with HCC and showed potential in assessing the degree of immune cell infiltration, informing clinical drug usage, and evaluating the effectiveness of immunotherapy. As a foundation for precise prognosis prediction in patients with HCC, the risk score represents a novel quantitative evaluation metric.

## Supporting information

S1 File (ZIP)

S1 Fig The basic information of all individual single-cell samples and the specific details before and after quality control. (TIF)

S2 Fig The 8 cell subclusters were annotated using classic immune cell markers. (TIF)

S3 Fig Colony formation and Transwell assays to evaluate SK-HEP-1 cell proliferation, migration, and invasion. (TIF)
