# Supplementary figures and images for: Identification of a senescence-related transcriptional signature to uncover molecular subtypes and key genes in hepatocellular carcinoma
Source: PLoS One. 2024 Oct 9;19(10):e0311696. doi: 10.1371/journal.pone.0311696 (PMC11463828; doi:10.1371/journal.pone.0311696)

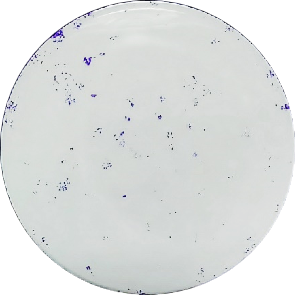

Supplement: S1 File — (ZIP) [file pone.0311696.s001.zip › Rawdata of WB and R script for manuscript/Colony formation/Colony formation-control.tif]

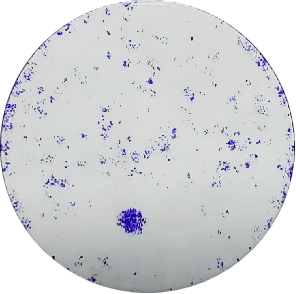

Supplement: S1 File — (ZIP) [file pone.0311696.s001.zip › Rawdata of WB and R script for manuscript/Colony formation/Colony formation-OE-G6PD.tif]

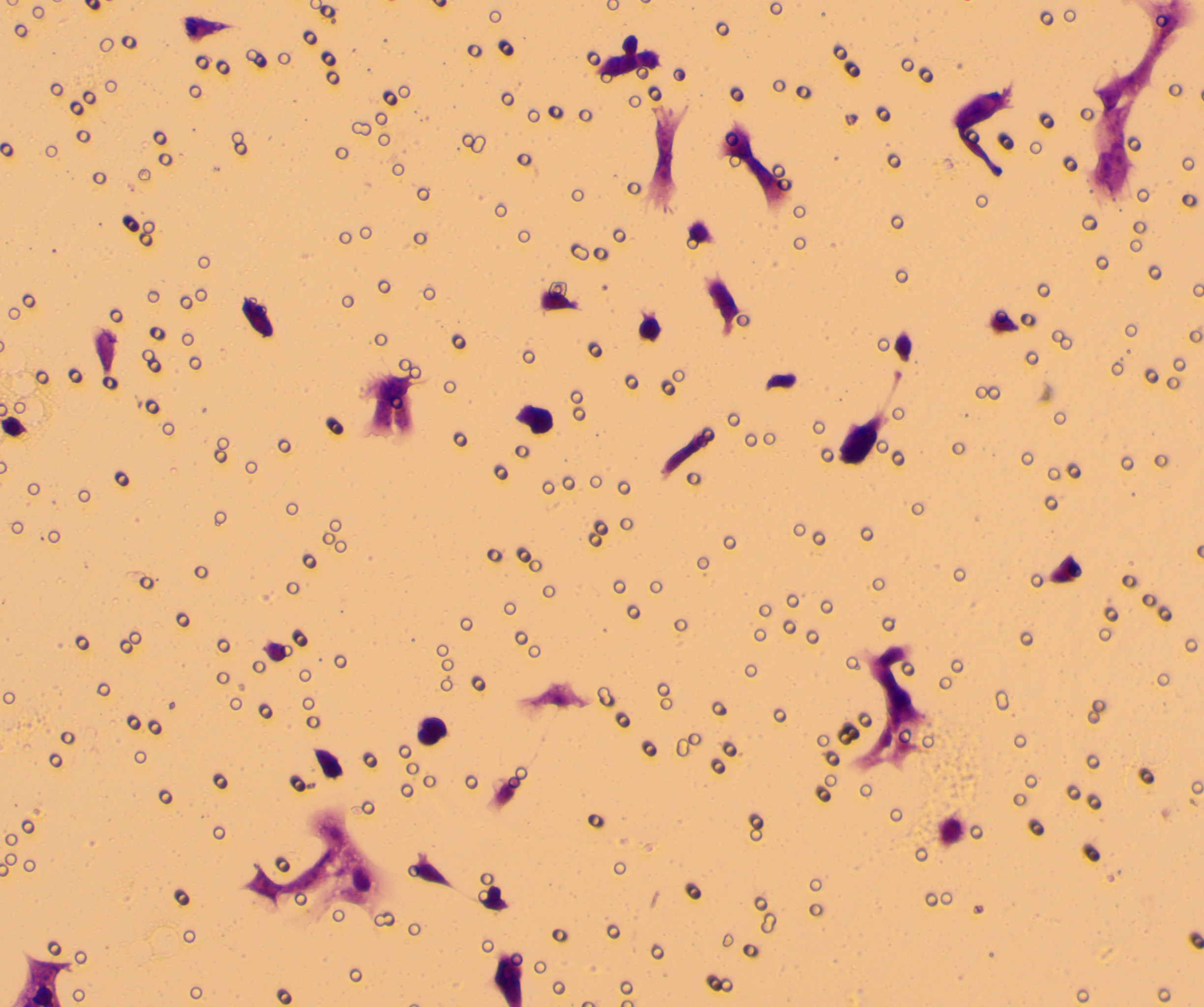

Supplement: S1 File — (ZIP) [file pone.0311696.s001.zip › Rawdata of WB and R script for manuscript/invasion/invasion-control.tif]

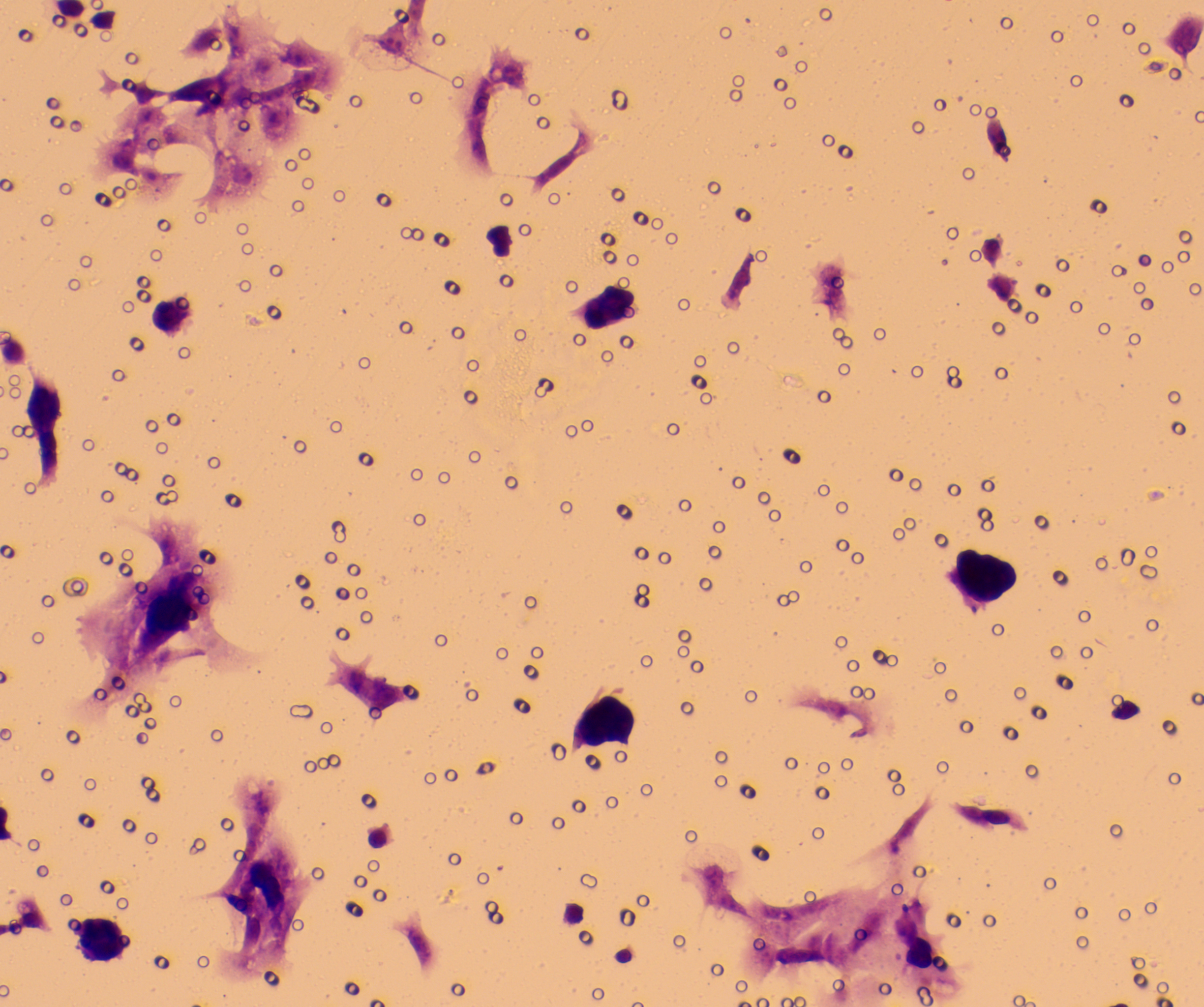

Supplement: S1 File — (ZIP) [file pone.0311696.s001.zip › Rawdata of WB and R script for manuscript/invasion/invasion-OE-G6PD.tif]

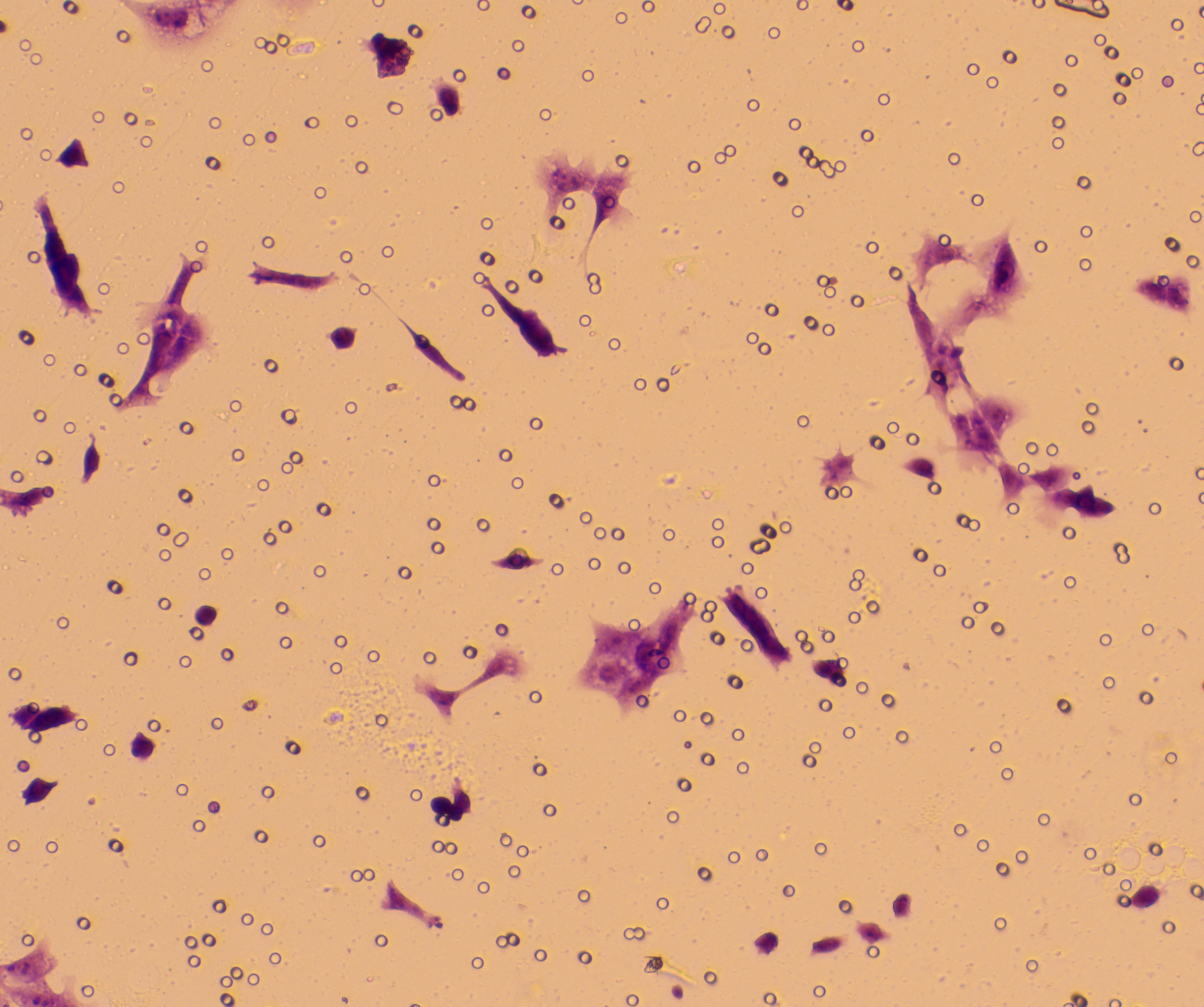

Supplement: S1 File — (ZIP) [file pone.0311696.s001.zip › Rawdata of WB and R script for manuscript/migration/migration-control.tif]

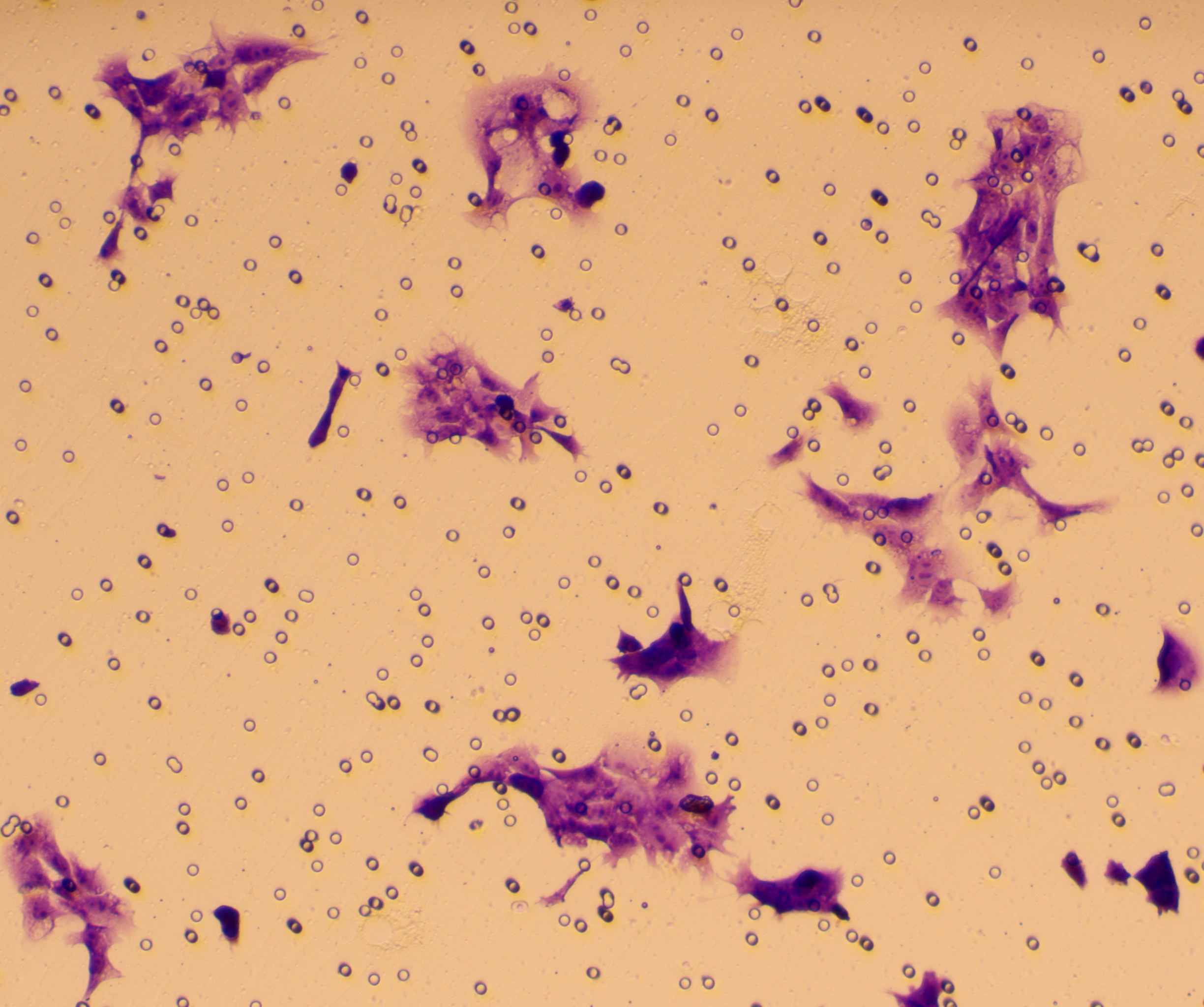

Supplement: S1 File — (ZIP) [file pone.0311696.s001.zip › Rawdata of WB and R script for manuscript/migration/migration-OE-G6PD.tif]

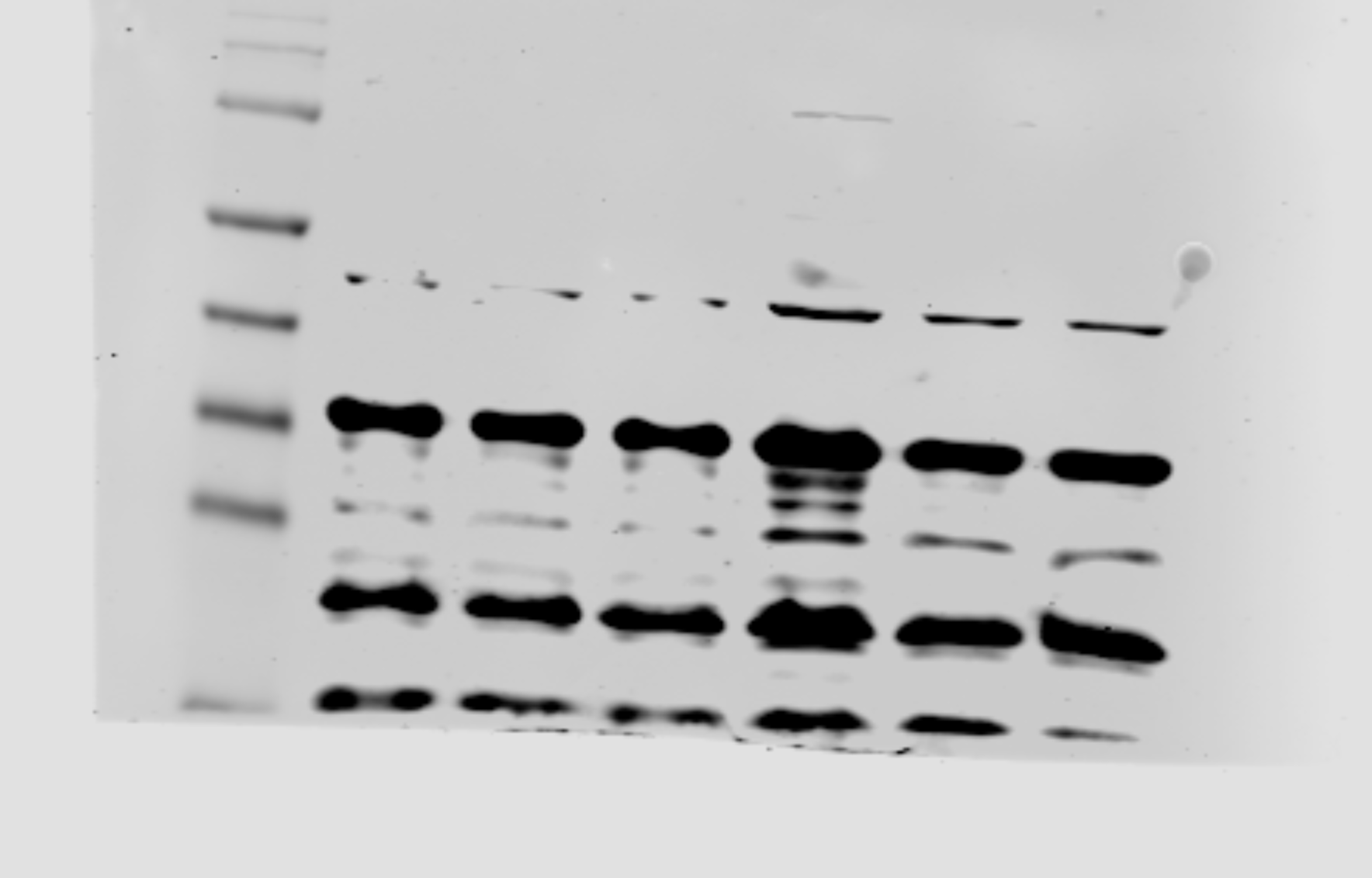

Supplement: S1 File — (ZIP) [file pone.0311696.s001.zip › Rawdata of WB and R script for manuscript/western blot/G6PD.tif]

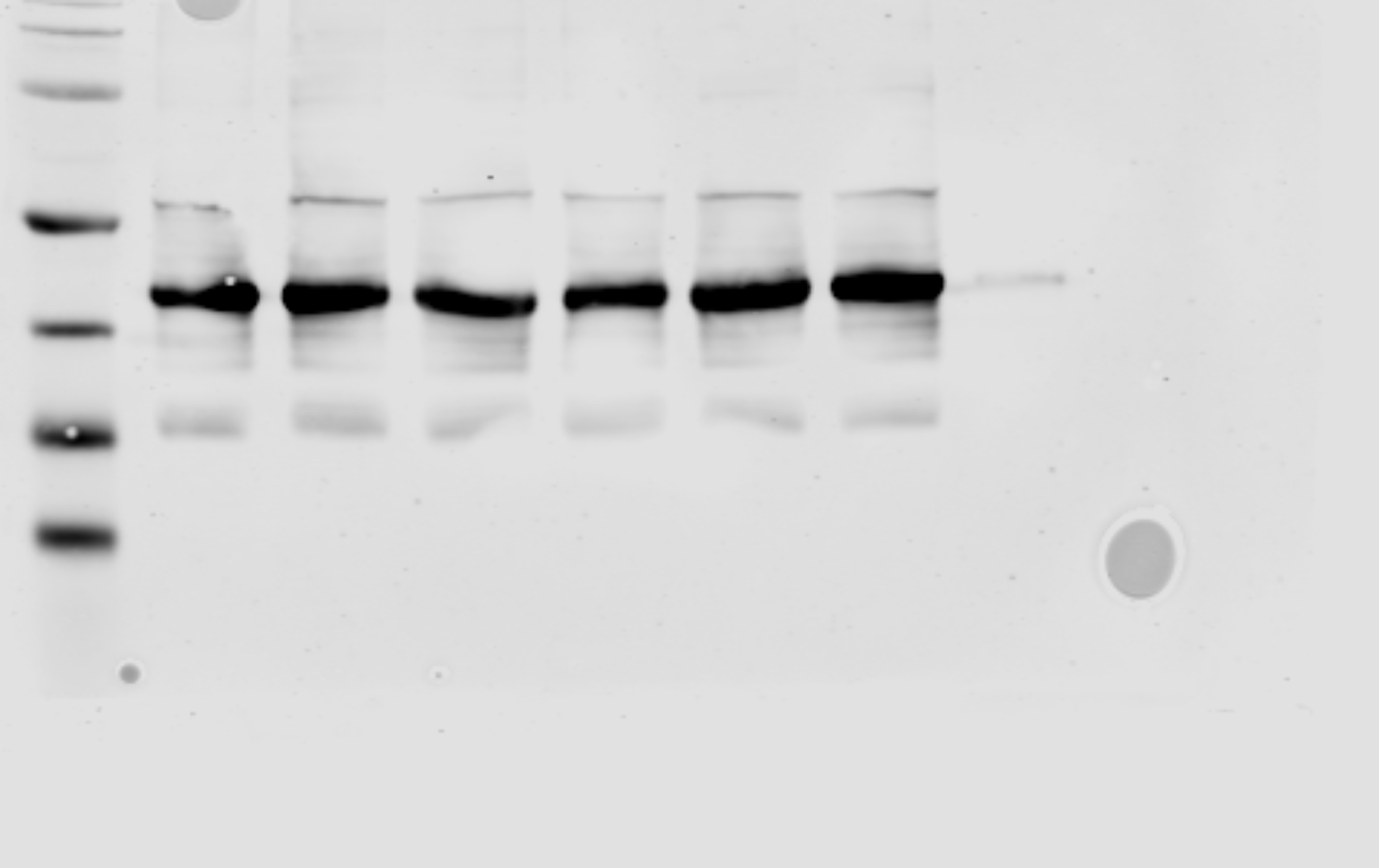

Supplement: S1 File — (ZIP) [file pone.0311696.s001.zip › Rawdata of WB and R script for manuscript/western blot/GAPDH.tif]

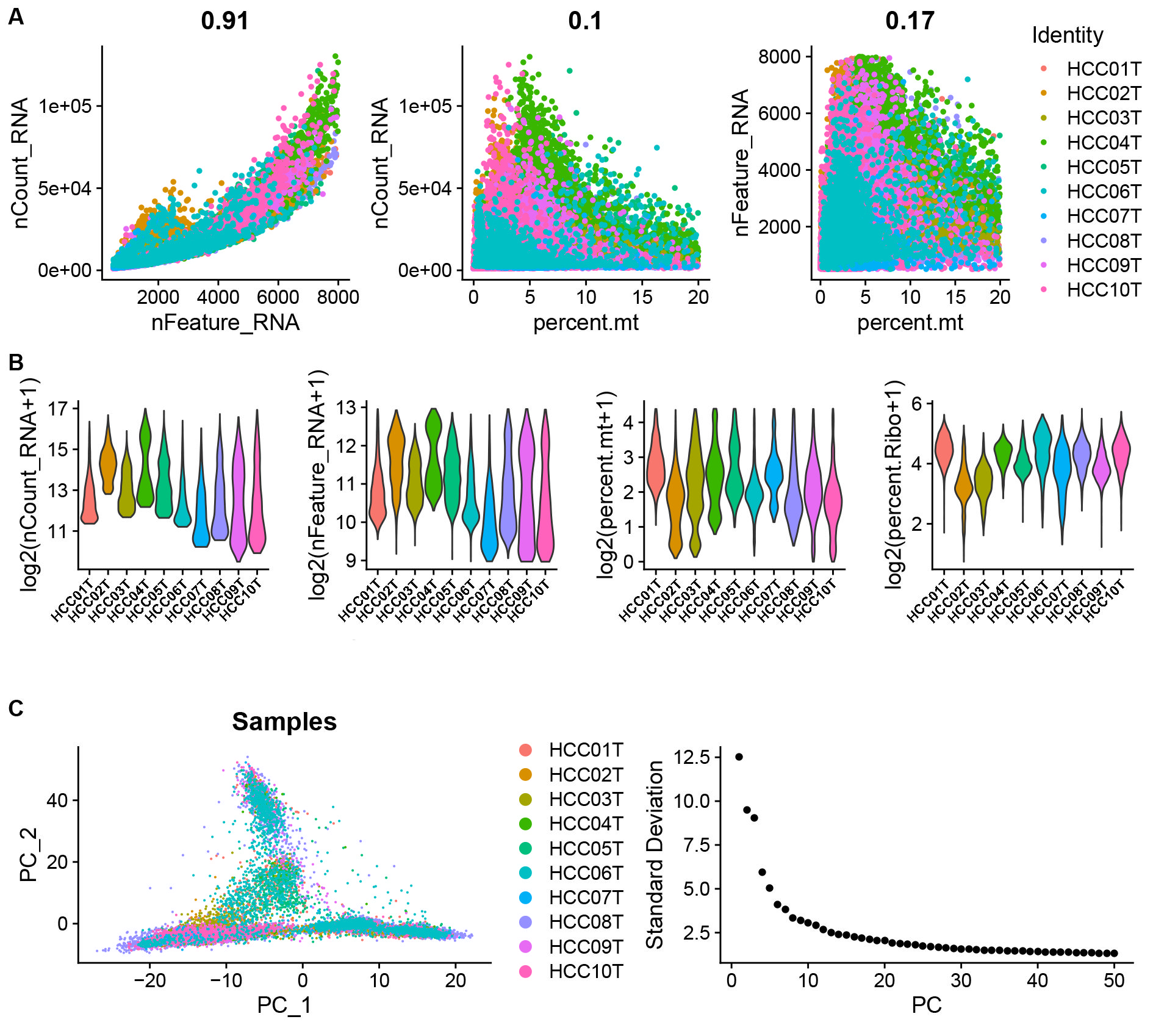

Supplement: S1 Fig — (TIF) [file pone.0311696.s002.tif]

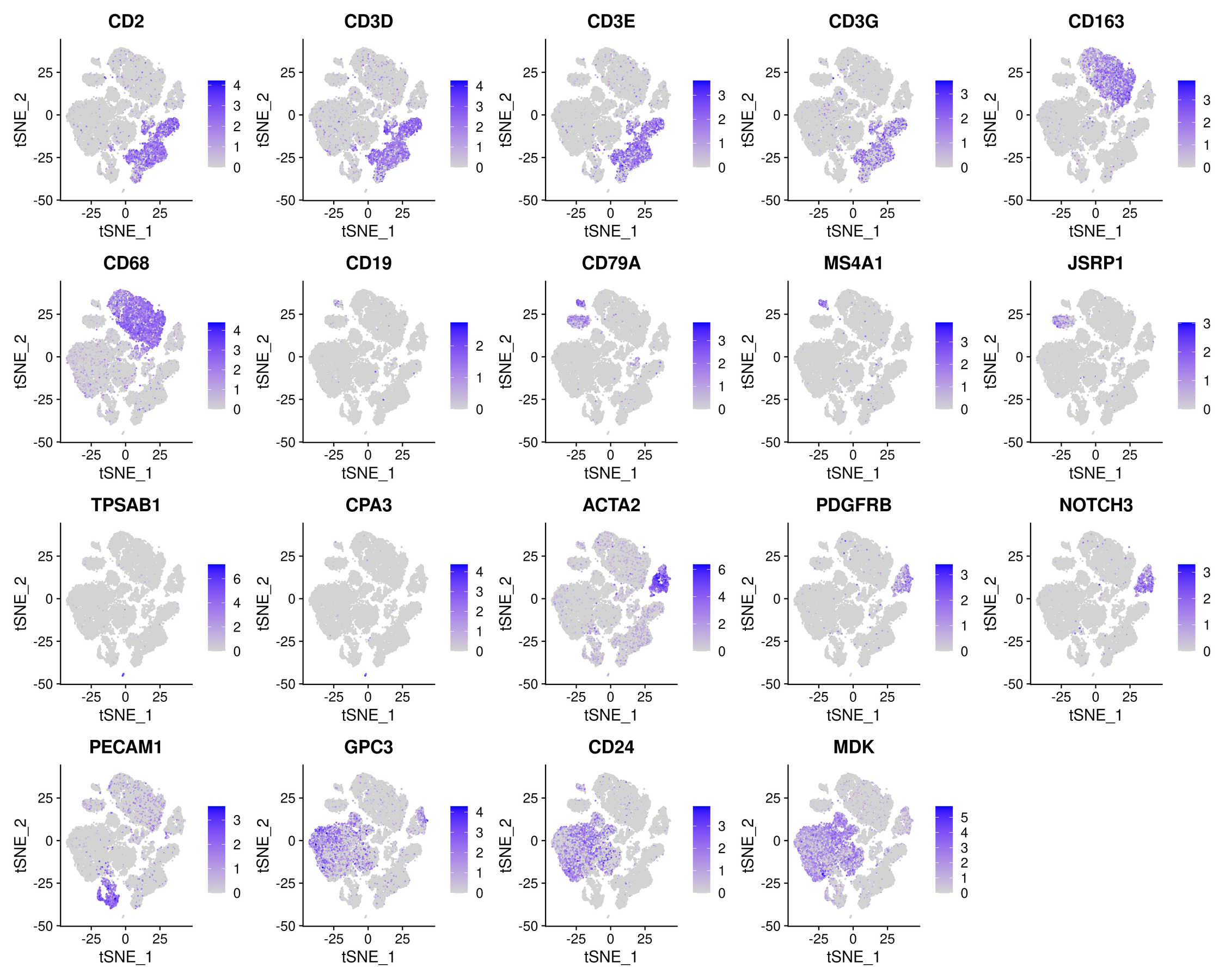

Supplement: S2 Fig — (TIF) [file pone.0311696.s003.tif]

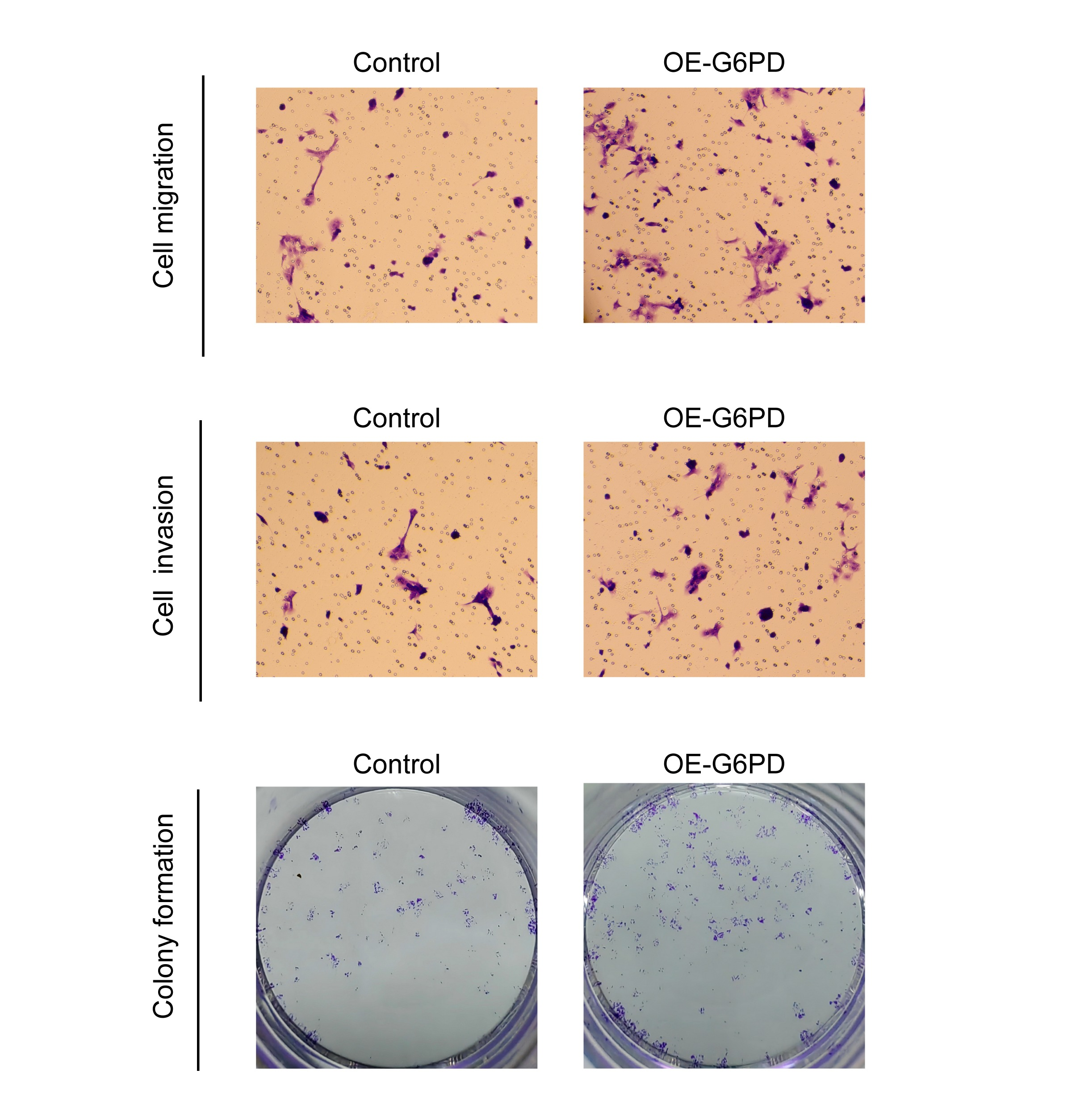

Supplement: S3 Fig — (TIF) [file pone.0311696.s004.tif]
